# Structure‐based pharmacophore modeling for precision inhibition of mutant ESR2 in breast cancer: A systematic computational approach

**DOI:** 10.1002/cam4.70074

**Published:** 2024-08-05

**Authors:** Sirajul Islam, Md. Al Amin, Kannan R.R. Rengasamy, A. K. M. Mohiuddin, Shahin Mahmud

**Affiliations:** ^1^ Department of Biotechnology and Genetic Engineering Mawlana Bhashani Science and Technology University Santosh Tangail 1902 Bangladesh; ^2^ Laboratory of Natural Products and Medicinal Chemistry (LNPMC), Center for Global Health Research, Saveetha Medical College and Hospital Saveetha Institute of Medical and Technical Sciences (SIMATS) Thandalam Chennai 602105 India

**Keywords:** breast cancer, estrogen receptor beta, MD simulations, mutant ESR2, structure based drug design, structure based pharmacophore modeling, ZINCPharmer

## Abstract

**Background:**

Breast cancer, a leading cause of female mortality, is closely linked to mutations in estrogen receptor beta (ESR2), particularly in the ligand‐binding domain, which contributed to altered signaling pathways and uncontrolled cell growth.

**Objectives/Aims:**

This study investigates the molecular and structural aspects of ESR2 mutant proteins to identify shared pharmacophoric regions of ESR2 mutant proteins and potential therapeutic targets aligned within the pharmacophore model.

**Methods:**

This study was initiated by establishing a common pharmacophore model among three mutant ESR2 proteins (PDB ID: 2FSZ, 7XVZ, and 7XWR). The generated shared feature pharmacophore (SFP) includes four primary binding interactions: Hydrogen bond donors (HBD), hydrogen bond acceptors (HBA), hydrophobic interactions (HPho), and Aromatic interactions (Ar), along with halogen bond donors (XBD) and totalling 11 features (HBD: 2, HBA: 3, HPho: 3, Ar: 2, XBD: 1). By employing an in‐house Python script, these 11 features distributed into 336 combinations, which were used as query to isolate a drug library of 41,248 compounds and subjected to virtual screening through the generated SFP.

**Results:**

The virtual screening demonstrated 33 hits showing potential pharmacophoric fit scores and low RMSD value. The top four compounds: ZINC94272748, ZINC79046938, ZINC05925939, and ZINC59928516 showed a fit score of more than 86% and satisfied the Lipinski rule of five. These four compounds and a control underwent molecular (XP Glide mode) docking analysis against wild‐type ESR2 protein (PDB ID: 1QKM), resulting in binding affinity of −8.26, −5.73, −10.80, and −8.42 kcal/mol, respectively, along with the control −7.2 kcal/mol. Furthermore, the stability of the selected candidates was determined through molecular dynamics (MD) simulations of 200 ns and MM‐GBSA analysis.

**Conclusion:**

Based on MD simulations and MM‐GBSA analysis, our study identified ZINC05925939 as a promising ESR2 inhibitor among the top four hits. However, it is essential to conduct further wet lab evaluation to assess its efficacy.

## 1 INTRODUCTION

Breast cancer stands as a pervasive global health concern, boasting an annual incidence surpassing 1.3 million cases and ranking among the leading causes of mortality, constituting over 23% of malignancies among women.^1^ Despite substantial strides in early detection and treatment modalities, breast carcinoma remains a formidable challenge, persistently exerting a significant toll on women's health worldwide.^2^ Surgical intervention, often followed by adjuvant radiation, chemotherapy, and endocrine therapy, constitutes the conventional approach, yet the complexity of breast cancer demands nuanced strategies, particularly in cases where resistance to estrogen receptor (ER) inhibition emerges during metastatic progression.^3^

Approximately 70% of breast cancers exhibit mutations in the ER, a pivotal element in the intricate web of endocrine resistance mechanisms.^4^ The ligand‐activated transcription factors, ERs, undergo conformational changes upon ligand binding, orchestrating the activation of target genes.^5^, ^6^ Notably, mutations in the estrogen receptor beta (ESR2) further complicate matters, with mutations occurring in the ligand‐binding domain presenting formidable challenges in endocrine therapy.^2^, ^6^ While hormone therapy has proven effective initially, long‐term exposure often leads to resistance, necessitating the development of novel drugs targeting ESR2 mutations.^7^ However, pharmacophore modeling is a crucial part of rational drug design,^8^ and emerges as a powerful tool in our exploration of phytochemical‐ER interactions. By identifying common structural features essential for biological activity, pharmacophore modeling aids in rationalizing the bioactivity of diverse compounds and streamlining the drug discovery process.^9^ The significance of pharmacophore modeling lies in its ability to unveil the essential molecular characteristics responsible for the specific biological activity.^10^ Subsequently, virtual screening is a computational technique that allows for the in‐silico evaluation of the binding affinity between the identified pharmacophore and the targeted receptors.^11^ Virtual screening accelerates the identification of lead compounds by prioritizing those with the highest predicted binding affinities for experimental validation.^12^ This efficient and cost‐effective approach optimizes resources and enhances the likelihood of identifying compounds with therapeutic potential.^13^, ^14^

In the current landscape of drug discovery, pharmacophore approaches have emerged as indispensable tools, integrating ligand‐based and structure‐based strategies to refine pharmacophore models, crucial in virtual screening, de novo design, and lead optimization. Virtual screening, a computational mainstay, probes vast ligand libraries for potential binding to target proteins, while molecular docking predicts molecular orientations within stable complexes.^15^ In this context, our study adopts a systematic computational approach to unravel the molecular and structural nuances of estrogen receptor beta (ESR2) mutant proteins, specifically within the ligand‐binding domain. Through pharmacophore modeling and virtual screening, we aim to identify shared pharmacophoric regions, paving the way for precision inhibition and the development of promising therapeutic targets against mutant ESR2 in breast cancer.

## 2 METHODS AND MATERIALS

### 2.1 Retrieval of the structures of ESR2 wild‐type and mutant proteins

**FIGURE 1:**
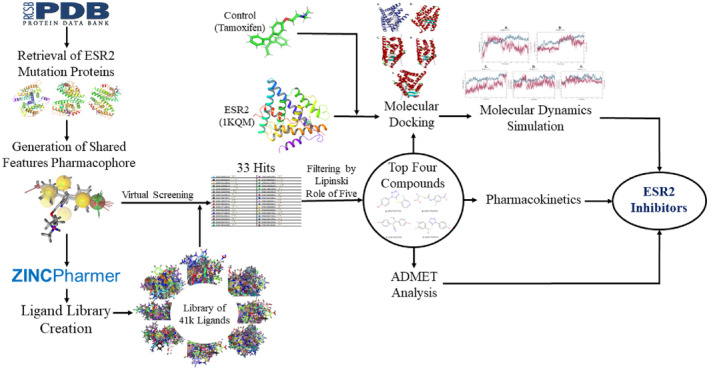
Overview of this study: From data collection, analysis, and drug development.

The retrieval of estrogen receptor beta wild‐type and mutant protein structures was conducted through a systematic search on the Protein Data Bank (PDB).^16^ The following criteria were employed to ensure the selection of high‐quality structural data‐ Scientific Name of Source Organism: Homo sapiens, Taxonomy: Eukaryota, Experimental Method: x‐ray diffraction,^17^ Refinement Resolution (Å): 2.0–2.5, Retrieval of ESR2 Wild and Mutant Proteins from Protein Data Bank (PDB) database.^16^ The search was initiated with these specific filters applied to the PDB database, focusing on structures derived from Homo sapiens (human) as the source organism, ensuring the reliability of the biological relevance. Eukaryota taxonomy was selected to narrow down the search to proteins of eukaryotic origin. The experimental method was specified as x‐ray diffraction to retrieve structures determined using this high‐resolution technique. Refinement resolution criteria were set within the range of 2.0–2.5 Å, aiming to obtain structures with optimal resolution for detailed structural analyses. The collected structures were subsequently assessed for their quality and adherence to the specified criteria. Any redundant or structurally incomplete entries were carefully excluded from the dataset to ensure the reliability of the ensuing analyses. However, Figure 1 illustrates the overview and workflow of this study.

### 2.2 Generation of shared feature pharmacophore model of ESR2 mutant proteins

We generated the shared feature pharmacophore (SFP) of ESR2 mutant proteins by using LigandScout software.^18^ Initially, individual pharmacophores were constructed for each co‐crystalized ligand of the protein‐ligand complex using the structure‐based pharmacophore^19^ (SBP) module to identify key pharmacophoric features, including hydrogen bond donors (HBD), acceptors (HBA), hydrophobic regions (HyPho), and aromatic moieties (Ar). During this process, specific attention was given to selecting pockets where the mutations occurred, ensuring a focused representation of crucial ligand‐binding interactions in that pocket. Subsequently, these individual pharmacophores were incorporated into the Alignment purpose to generate the shared feature pharmacophore (SFP) model. Finally, the SFP model was constructed by combining the individual pharmacophores. The resulting SFP for ESR2 mutant proteins provides a consolidated and insightful representation of key ligand recognition patterns, serving as a valuable tool for subsequent virtual screening and elucidation of ligand interactions within the context of ESR2 mutants.

### 2.3 Ligand library creation

After aligning the separated pharmacophore map of mutant proteins, the SFP model has identified with the following features (Figure S1, Table 2): 3 Hydrophobic: H1, H2, H3; 1 Halogen bond donor: XBD; 3 Hydrogen bond acceptor: HBA1, HBA2, HBA3; 2 Hydrogen bond donor: HBD1 and HBD2; 2 Aromatic: Ar1, Ar2. At this point, the next goal is to use the 11 identified features to generate a drug database from ZINCpharmer. ZINCpharmer can create a more specific drug library by using only 5–6 features (like HBD, HBA, Ar, and HyPho) at a time as query features. Since we have a total of 11 features, we used an in‐house Python script to distribute the features using a permutation formula. As a result, we determined the possible combinations and explored the possible outcomes with the help of in‐house Python scripts.^20^ Finally, by using these combinations as query features, we created a ligand library by 1st round of screening from the ZINCPharmer database.^21^ However, one of the major features of drugs is aromatic binding, along with HBA and HBD, which are crucial for smooth interaction with the active site of the protein. Hydrophobic interactions are more complex within the cellular system, so we allow this binding to be managed by the default parameters of the search tool. Here is the five feature permutation (where three features i.e., HBD, HBA, and Ar are constant) process‐ The total number of unique configurations is calculated using the formula:

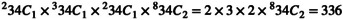




This formula represents the selection of 1 aromatic from 2 (2C1), 1 hydrogen bond acceptor from 3 (3C1), 1 hydrogen bond donor from 2 (2C1), and determining the remaining 2 features from the remaining 8 (8C2). The result is 336 distinct molecular groups.

### 2.4 Virtual screening of SFP model

In this study, second round of virtual screening was conducted using LigandScout software to identify potential lead compounds from our ligand library.^18^ Our previously generated SFP model was employed for virtual screening against our ligand database. Ligands were prioritized based on their ability to match the identified pharmacophoric features, and ranking was performed according to fit scores, indicating potential favorable interactions with the target.

### 2.5 Refinement of potential hit compounds according to Lipinski rule of five

For the refinement of potential hit compounds, a crucial step involved the application of the Lipinski rule of five, a set of criteria designed to assess the drug‐likeness and oral bioavailability of small molecules. To execute this filtering process, all 33 hit compounds (obtained their sdf format from the ZINCPharmer database), were subjected to the Lipinski rule of five^22^ analysis using the PyRx software.^23^ The procedure began by launching the PyRx tool and loading the ligands in sdf format, ensuring compatibility with the software. Subsequently, the filter option was accessed within PyRx, initiating the Lipinski rule of five filter selection. The software then applied the Lipinski criteria, which include parameters such as molecular weight, lipophilicity (expressed as LogP), number of hydrogen bond donors, and number of hydrogen bond acceptors (HBAs). Upon completion of the filtering process, compounds that met the Lipinski Rule of Five criteria were identified and collected as the filtered results. This step aimed to prioritize compounds with favorable physicochemical properties, enhancing their potential for successful drug development.

### 2.6 Physicochemical properties (pharmacokinetics) analysis

The analysis of the physicochemical properties of the ligands was conducted using the SwissADME web server.^24^ This online tool was employed to assess various key characteristics essential for drug‐likeness. The methodology involved submitting the chemical structures of the ligands to the SwissADME server, which then calculated and provided information on important physicochemical parameters. These parameters included Lipinski's rule of five, a compound is considered more likely to exhibit drug‐like properties if it satisfies at least four out of five criteria: a molecular weight of 500 Daltons, a hydrogen bond acceptor ≤5, a hydrogen bond donor ≤10, a lipophilicity <5, TPSA (topological polar surface area) 20–130 Å2, and a molar refractivity range from 40 and 130,^25^ which offering determination of drug‐like properties and aiding in the selection of promising candidates for further drug development.

### 2.7 In silico approach to structure‐based drug design

#### 2.7.1 Molecular docking analysis

Molecular docking, a pivotal computational technique in drug discovery, was conducted to explore the binding interactions between the top four compounds and the ESR2 (1KQM) protein. This analysis was executed employing the Glide Mode of Maestro version 13.7, a comprehensive software package developed by Schrödinger LLC.^26^, ^27^, ^28^, ^29^

The first step involved the preparation of the ligands and the target protein. The top four ligands were incorporated into the workstation, and the energy was minimized using the OPLS3e (optimized potentials for liquid simulations) force field in the Ligprep module of the software to ensure proper geometry, energy minimization, and optimization of their conformations.^30^ The generated output file (Best conformations of the ligands) was further used for docking study. Simultaneously, the ESR2 protein structure (PDB ID: 1KQM) was prepared by Protein Preparation Wizard,^31^ including the addition of hydrogen atoms, refinement of bond orders, optimization of side‐chain conformations, and assigning the charges. Generated Het states using Epik at pH 7.0 ± 2.0. The protein was modified by analyzing the workspace water molecules and others. The critical water molecules remained the same, and the rest of the molecules apart from the heteroatoms were deleted. Finally, the protein was minimized using the OPLS‐3 force field.^32^ A grid was created by considering the co‐crystal ligand, which was included in the active site of the selected protein target.

Subsequently, the prepared ligands were docked with the ESR2 protein using the Glide Mode of Maestro. Glide employs an accurate and efficient algorithm to predict ligand binding conformations and binding affinities. The docking simulations explored potential binding modes and energetically favorable poses of the ligands within the protein's binding site.

#### 2.7.2 Molecular dynamics simulation of the selected protein‐ligand complexes

Molecular dynamics (MD) simulation is usually conducted to inspect the stability of the complex of candidate drug compounds and the target protein.^33^, ^34^, ^35^ We conducted MD simulations spanning a 200‐ns duration using the Desmond software package developed by Schrödinger LLC. Before initiating the simulations, the protein‐ligand complexes underwent preprocessing procedures, including optimization and minimization, using the Protein Preparation Wizard of Maestro software. System construction was facilitated with the System Builder tool. To emulate realistic environmental conditions, we adopted the TIP3P solvent model within an orthorhombic simulation box.^36^ The simulations were executed employing the OPLS_2005 force field, and the models were neutralized by the addition of counter ions when necessary.^35^ To replicate physiological conditions, we introduced a 0.15 M salt solution (NaCl). For the entirety of the simulation, the equilibrium was established using NVT and NPT ensembles, maintaining temperature and pressure values, and ensuring the conservation of moles (N), pressure (P), and temperature (T) at 300 K and 1 atm, respectively. Pre‐simulation relaxation procedures were carried out for the models. To evaluate simulation stability, we computed parameters such as the radius of gyration (RG), solvent‐accessible surface area (SASA), root mean square deviation (RMSD),^37^ root mean square fluctuations (RMSF), and Torsion angels^38^ for the control and top four selected complexes.

#### 2.7.3 MM‐GBSA analysis

The MM‐GBSA (molecular mechanics—generalized born surface area) analysis was performed to investigate the free binding energies by using the Glide pose viewer file of the top four lead compounds.^39^, ^40^, ^41^, ^42^, ^43^, ^44^, ^45^ The prime module of Schrödinger software was used to calculate the optimal binding energy of the selected top four complexes. For the analysis, the VSGB 2.1 model^46^ was exploited, having an S‐OPLS force field inclusive of an implicit solvent model in addition to physics‐based modifications for p–p interactions, hydrophobic interactions, and hydrogen bonding self‐contact interactions.^30^

The changes in free energy upon binding were calculated by using the following expressions:

$${\mathrm{\Delta G}}_{\text{Bind}}={\mathrm{\Delta G}}_{\text{complex}}-\left({\mathrm{\Delta G}}_{\text{protein}}+{\mathrm{\Delta G}}_{\text{ligand}}\right)$$

ΔG_Bind_ is the ligand binding energy, ΔG_complex_ is the energy of the complex, ΔG_protein_ is the energy of the receptor without the ligand, and ΔG_ligand_ is the energy of the unbound ligand.

#### 2.7.4 ADMET analysis

The ADMET analysis was conducted using the PKCSM server, a computational tool designed for the prediction of pharmacokinetic and toxicity properties of small molecules by submitting the chemical structures of the ligands to the PKCSM server.^47^, ^48^ The server utilizes a range of computational models and algorithms to predict various ADMET parameters, including absorption (Caco‐2 permeability, human intestinal absorption),^49^ distribution (blood–brain barrier permeability, central nervous system permeability), metabolism (CYP1A2 inhibition),^50^ excretion (total clearance), and toxicity (hERG inhibition, oral rat acute toxicity).^51^ The output provides valuable insights into the pharmacokinetic profile and potential toxicological risks associated with the ligands. The predictions contribute to the assessment of the ligands' drug‐likeness and suitability for further drug development.

## 3 RESULTS

### 3.1 ESR2 mutant proteins collection

**FIGURE 2:**
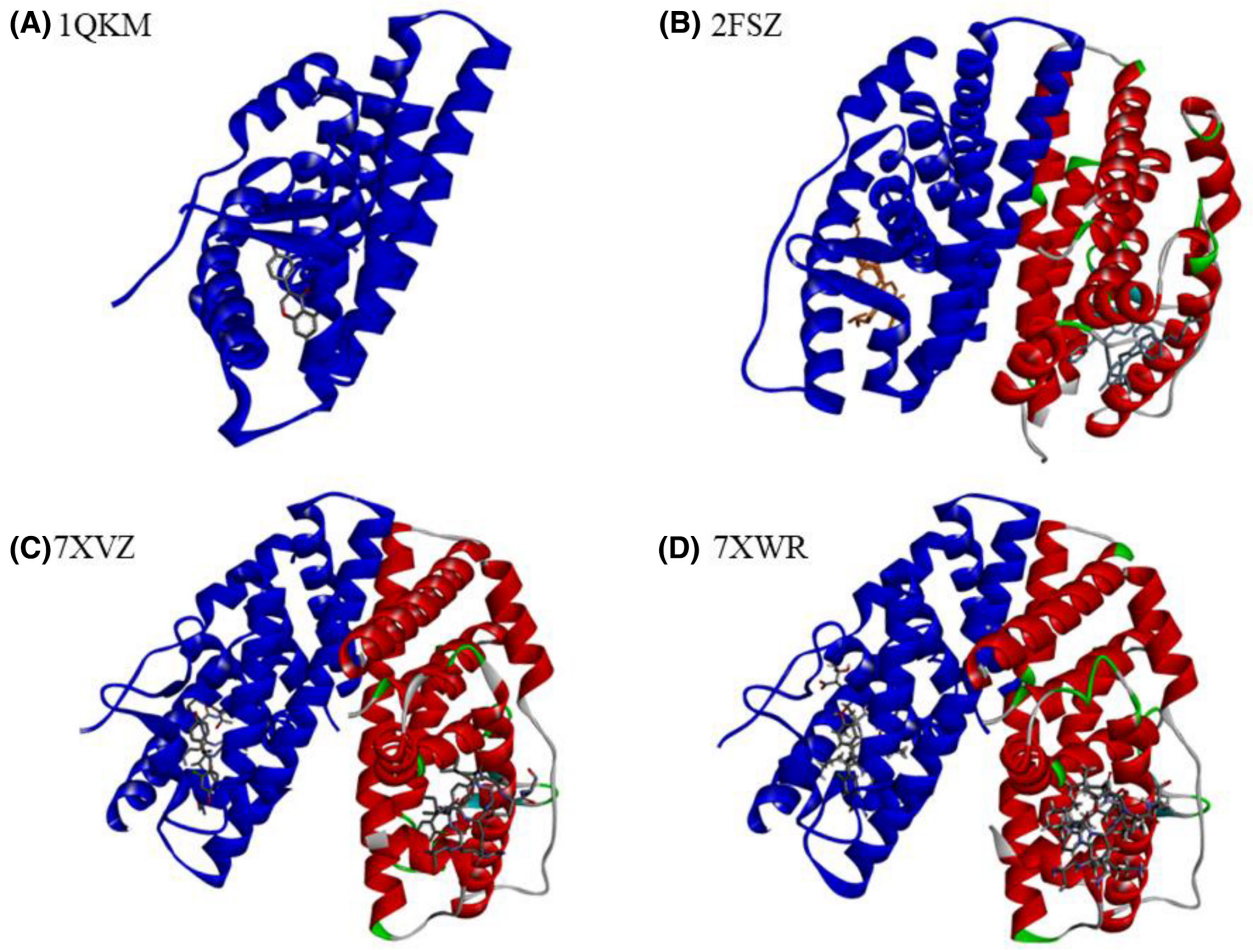
3D structures of ESR2 wild protein (A) and mutant proteins (B–D) with their co‐crystalized ligands.

**FIGURE 3:**
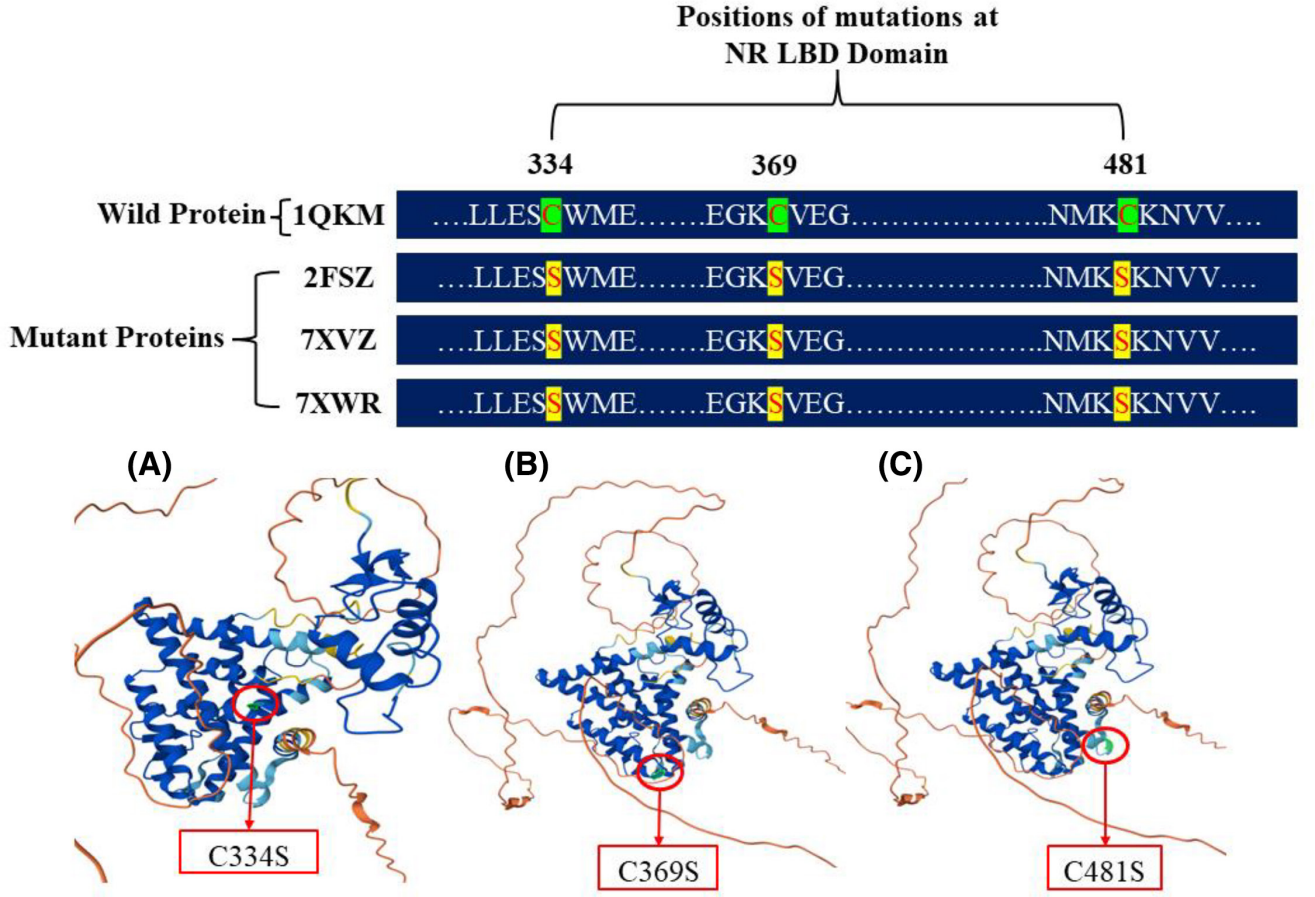
Positions of mutations in Ligand Binding Domain (LBD) (A) C334S (B) C369S (C) C481S of the mutant proteins 2FSZ, 7XVZ, and 7XWR.

The wild‐type ESR2 protein 1QKM (Figure 2A) comprises 255 amino acid residues in chain A. In contrast, the mutated ESR2 proteins, such as 2FSZ (Figure 2B), 7XVZ (Figure 2C), and 7XWR (Figure 2D) contain two chains, A (blue) and B (red). These chains are dimer of the wild‐type 1QKM protein monomer and each chain has a nearly similar number of amino acids as the 1QKM monomer protein. These three mutant proteins have similar mutations in the amino acid residues C334S, C369S, and C481S (According to the amino acid residue number of ESR2 protein: UniProt ID‐ Q92731). These structural differences serve as the foundation for subsequent pharmacophoric modeling and drug screening in our study. Summary of their features, 3D structures, and mutation positions of mutant ESR2 proteins were presented in Table 1, Figure 3 respectively.

**TABLE 1:** Features of ESR2 wild protein and mutant proteins.

SL	ESR2	Chain	Length (AA)	Mutations	Bound ligand/s	Ligands formula
01	1QKM	A	255	No	GENISTEIN	C_15_ H_10_ O_5_
02	2FSZ	A, B	246	C334S, C369S, C481S	4‐HYDROXYTAMOXIFEN	C_26_ H_29_ N O_2_
03	7XVZ		247		(2~{R})‐3‐(2‐chloranyl‐4‐oxidanyl‐phenyl)‐2‐(4‐hydroxyphenyl)propanenitrile	C_15_ H_12_ Cl N O_2_
04	7XWR		247		(2~{S})‐2‐(2‐chloranyl‐4‐oxidanyl‐phenyl)‐3‐(4‐hydroxyphenyl)propanenitrile	C_15_ H_12_ Cl N O_2_

### 3.2 Shared featured pharmacophore model generation

**FIGURE 4:**
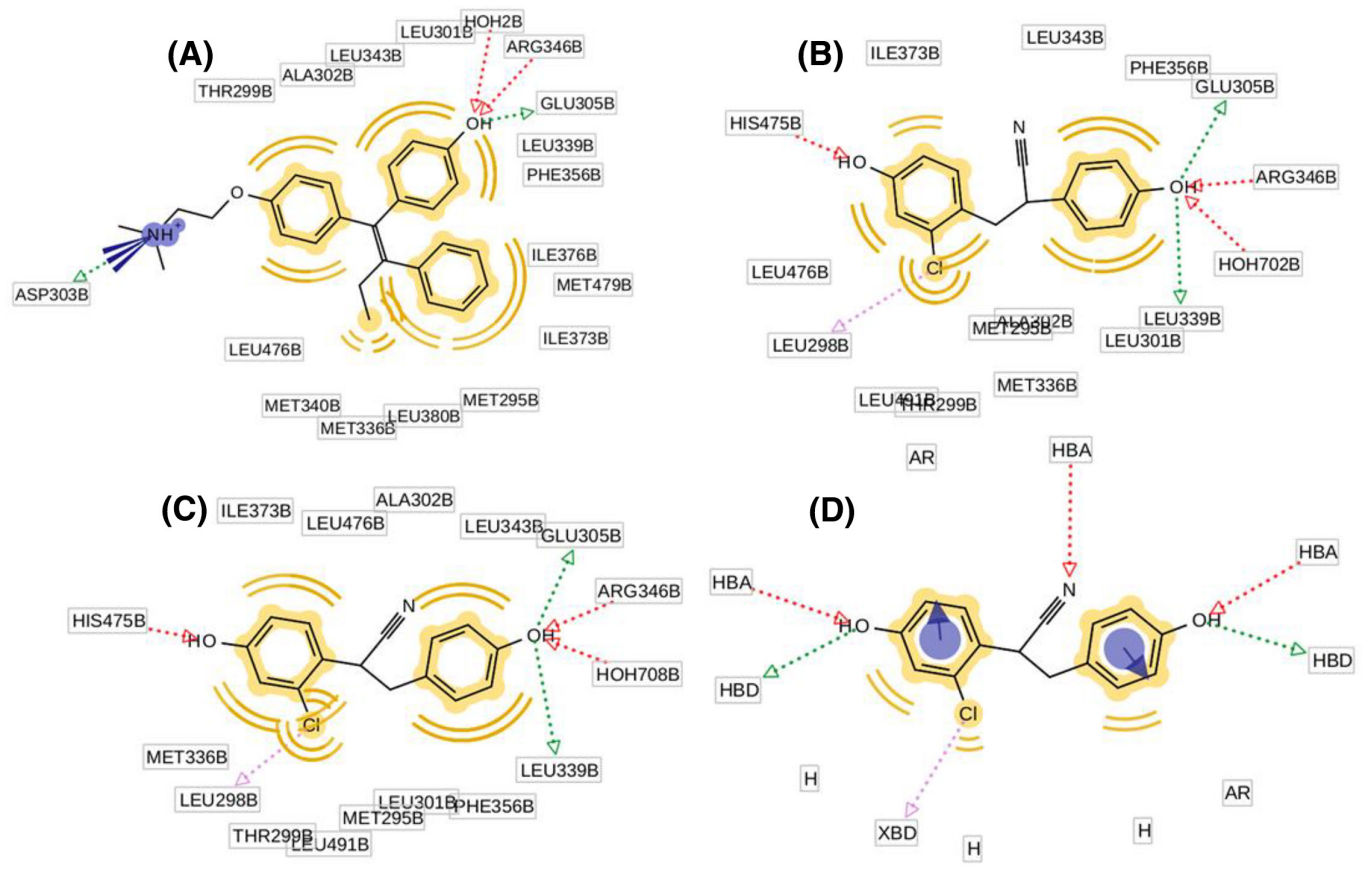
Individual pharmacophores of Co‐crystal ligands of mutant proteins (A) 2FSZ (B) 7XVZ (C) 7XWR and their (D) shared feature pharmacophore (SFP) model.

Pharmacophore analysis is integral to drug design, and in this study, it emerges as a crucial component for understanding the molecular characteristics of the ESR2 mutant proteins associated with breast cancer. Individual pharmacophore model of co‐crystalized ligands of the mutant proteins (Figure 4), utilizes red arrows to signify HBAs, green arrows for hydrogen bond donors (HBD), and yellow spheres for aromatic rings (Ar). Remarkably, all three pharmacophores share commonalities, containing essential features such as HBAs, hydrogen bond donors (HBD), and Ar. To consolidate these findings and identify shared features across the selected ESR2 mutant proteins, the pharmacophore models are aligned based on their structural attributes. This alignment results in the generation of a SFP model, depicted in Figure 4D, providing a comprehensive representation of the common molecular features critical for potential drug interactions within the context of breast cancer. The pharmacophoric features of wild‐type and mutant proteins are summarized in Table 2.

**TABLE 2:** Pharmacophoric features of 2FSZ, 7XVZ, 7XWR, and shared feature pharmacophore (SFP) model.

SL	ESR2	Pharmacophoric features
01	2FSZ	HBD: 2, HBA: 2, HPho: 9, Ar: 3
02	7XVZ	HBD: 2, HBA: 3, HPho: 7, Ar: 2, XBD: 1
03	7XWR	HBD: 2, HBA: 3, HPho: 5, Ar: 2, XBD: 1
04	SFP model	HBD: 2, HBA: 3, HPho: 3, Ar: 2, XBD: 1

### 3.3 Ligands library creation

By applying the formula of permutation, we found 336 combinations of the 11 pharmacophoric features of the SFP model and we explored the possible outcomes (Table S1) of the combinations with the help of Python. Finally, by using these combinations as query features, we created a ligand library of 41,248 compounds by 1st round of repeated screening from 21.777093 million compounds of the ZINCPharmer database.

### 3.4 Virtual screening and filtering by Lipinski rule of five

**FIGURE 5:**
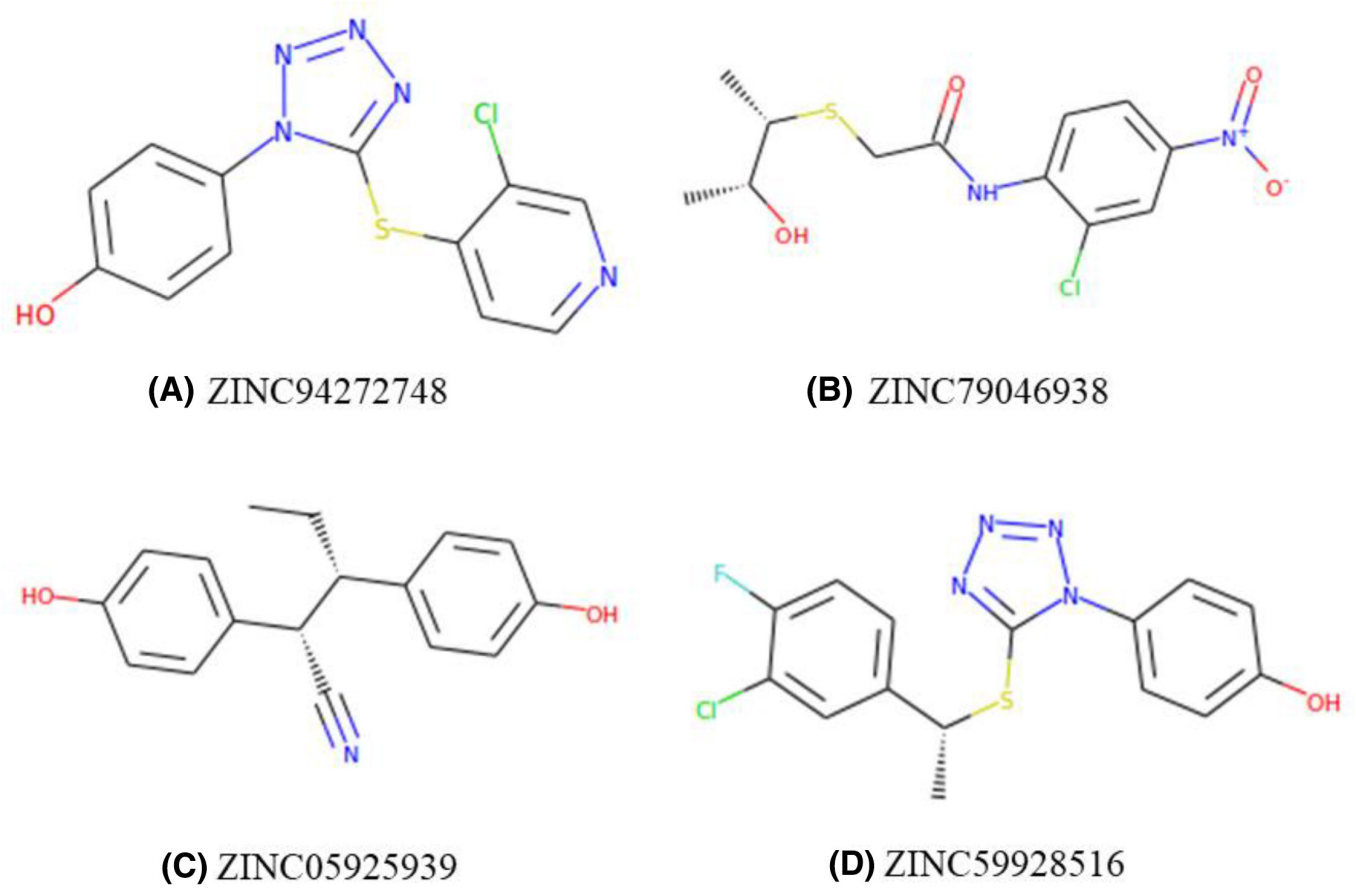
2D structures of the top four hit compounds (A–D) from the analysis.

**FIGURE 6:**
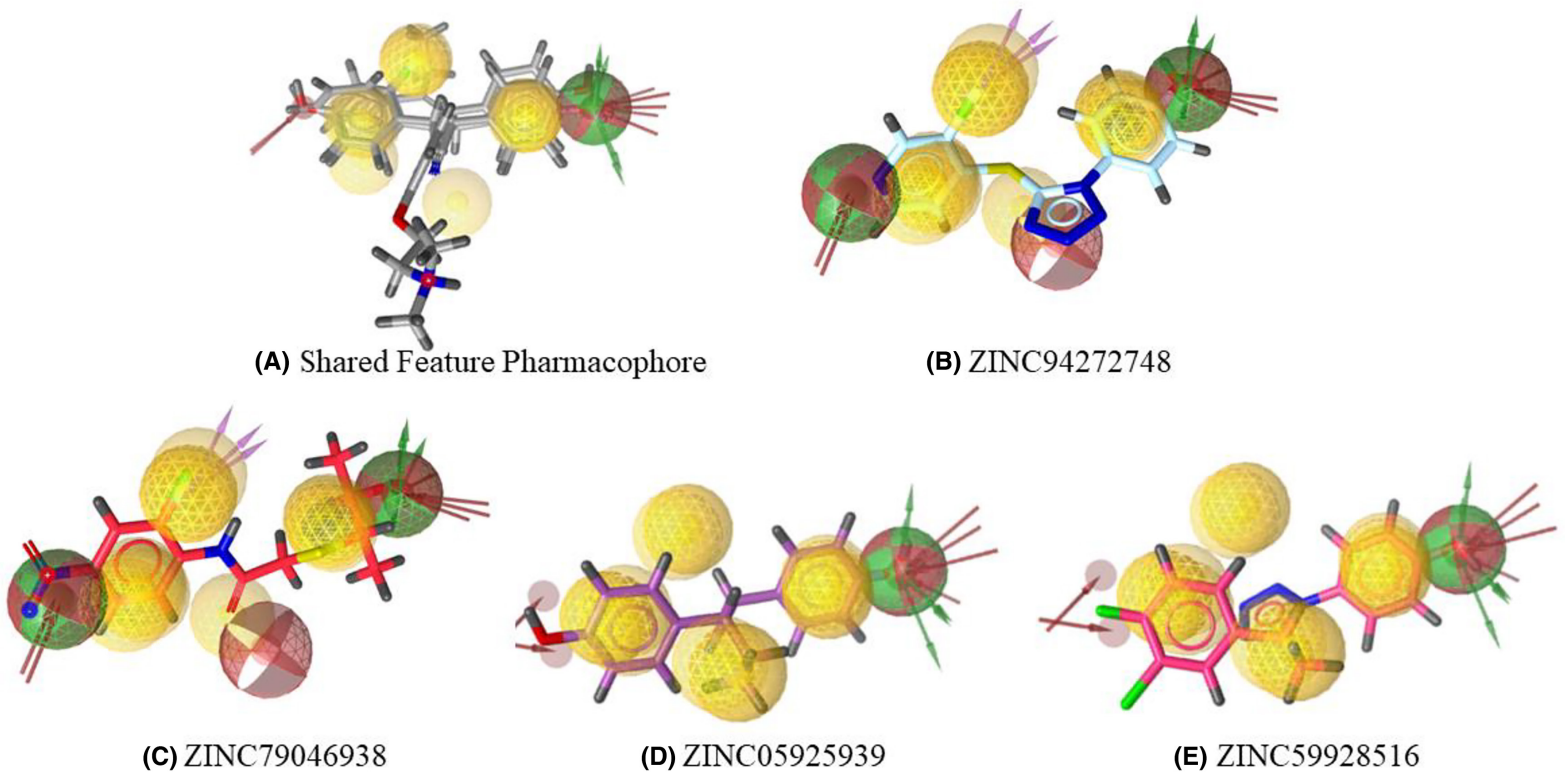
Individual pharmacophores (3D) of top four hit compounds (B–E) which show almost similar pharmacophoric features with (A) SFP Model.

**FIGURE 7:**
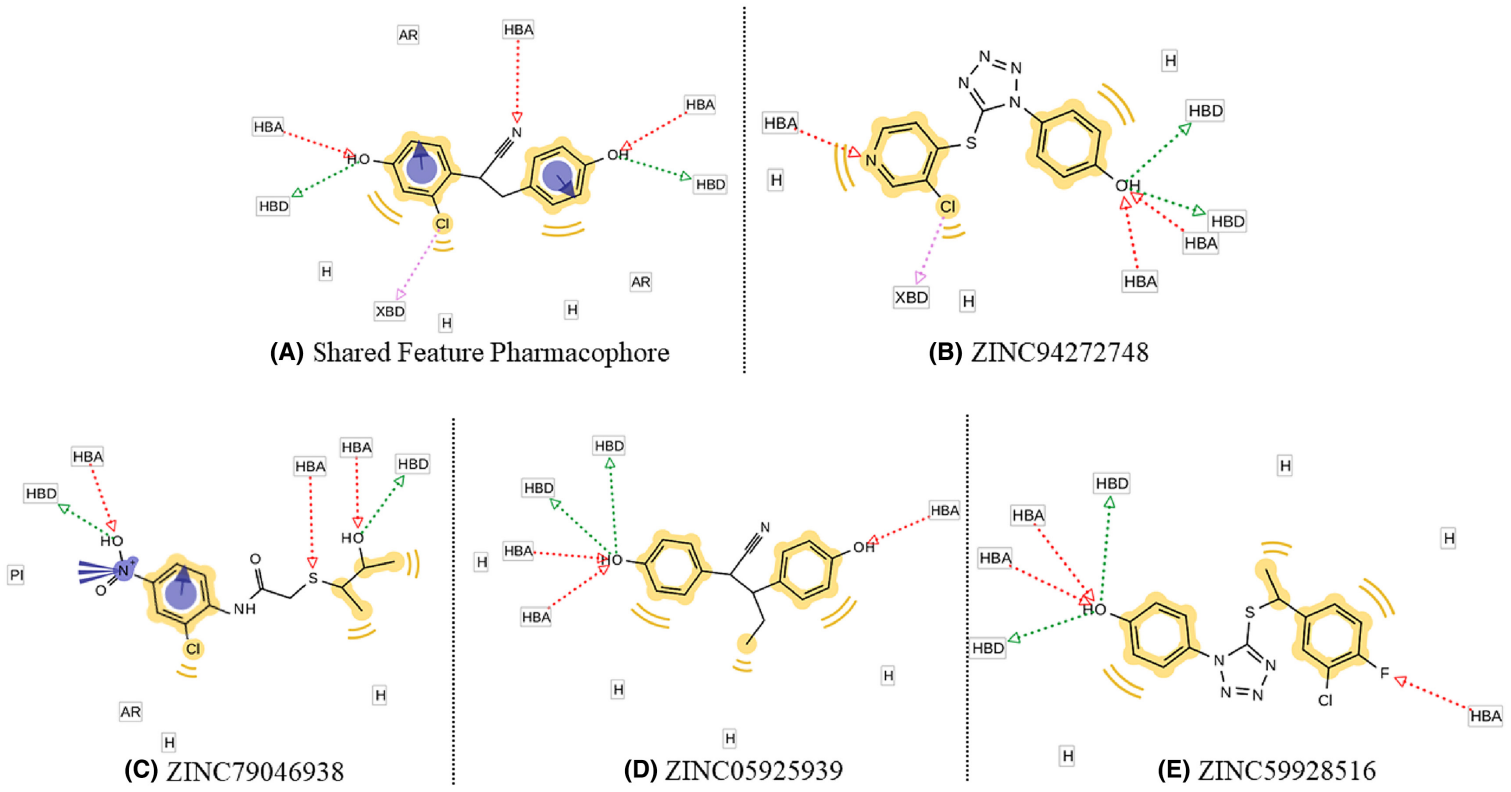
Individual pharmacophores (2D) of top four hit compounds (B–E) which show almost similar pharmacophoric features with (A) SFP Model.

In the 2nd round of virtual screening, 33 compounds (Table S2) closely aligned with the SFP were identified, boasting fit scores from 67.69 to 96.90. These scores quantitatively measure molecular correspondence, indicating significant alignment with pharmacophoric features crucial for drug interactions.^52^ Table 3 demonstrated the top four hit compounds with their hit scores and Figures 5, 6, 7 presented the 2D structures of the top four hit compounds and 3D and 2D visualizations of their pharmacophores respectively. Subsequently, the hit compounds underwent strict Lipinski rule of five evaluations, and all 33 met the criteria with some violations, confirming favorable physicochemical properties. The top four compounds were meticulously chosen for in‐depth analysis, ensuring alignment with the pharmacophore and adherence to essential drug‐like properties. This stringent selection establishes these compounds as promising candidates for breast cancer therapeutics.

**TABLE 3:** Top four hit compounds with their fit scores.

SL	Name	Pharmacophore fit score	Number of conformers	Matching features
01	ZINC94272748	96.90	25	9
02	ZINC79046938	95.91	25	9
03	ZINC05925939	87.55	11	8
04	ZINC59928516	86.96	25	8

### 3.5 Physicochemical properties analysis

The physicochemical properties of the ligands provide valuable insights into their drug‐likeness and pharmacokinetic profiles.^53^, ^54^, ^55^, ^56^ Tamoxifen, used as a control, has a molecular weight (MW) of 371.51 g/mol and exhibits low gastrointestinal (GI) absorption, with multiple violations in Lipinski, Ghose, Veber, Egan, and Muegge rules. ZINC94272748, ZINC79046938, ZINC05925939, and ZINC59928516 demonstrate improved drug‐likeness with MW values ranging from 267.32 to 350.80 g/mol. These ligands exhibit high GI absorption, adhering to Lipinski, Ghose, Veber, Egan, and Muegge criteria. Notably, ZINC94272748 has a lower lipophilicity (Consensus Log Po/w) of 2.32 compared to other ligands. All ligands have a negative water solubility (Log S), indicating low water solubility, which is a common characteristic in drug development. The MR values suggest differences in the size and polarizability of the ligands, with ZINC59928516 having the highest MR. The number of HBAs and donors, rotatable bonds, and surface area values contribute to the ligands' pharmacokinetic profiles. None of the ligands are predicted to be blood–brain barrier (BBB) permeant. Overall, the ligands, especially ZINC94272748, ZINC79046938, ZINC05925939, and ZINC59928516, exhibit favorable drug‐like properties and GI absorption, making them promising candidates for further investigation in drug development (Table S3).

### 3.6 Molecular docking analysis

**FIGURE 8:**
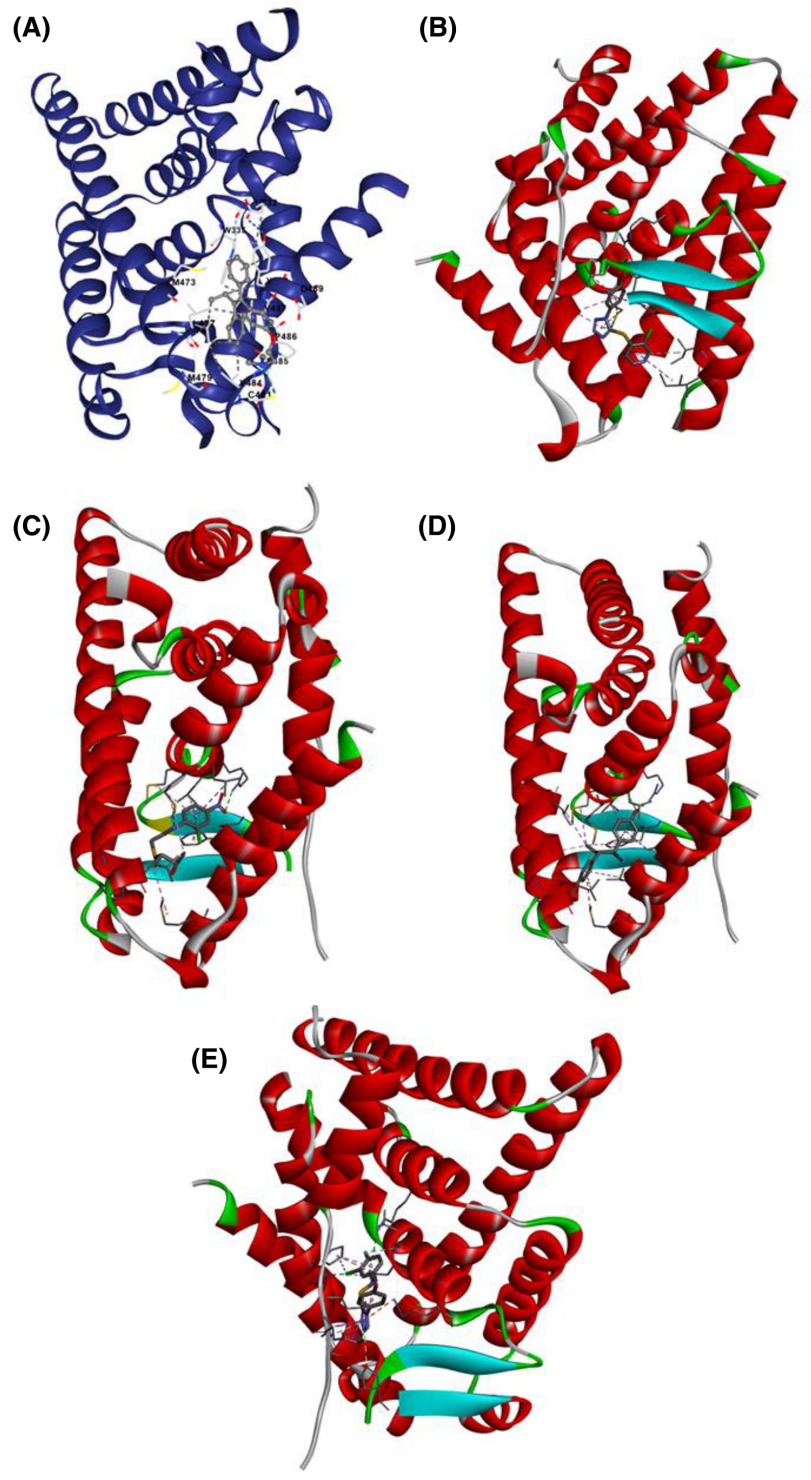
3D Complexes of molecular docking analysis of (A) Control (Tamoxifen), (B) ZINC94272748, (C) ZINC79046938, (D) ZINC05925939, and (E) ZINC59928516 with ESR2 protein 1QKM.

**FIGURE 9:**
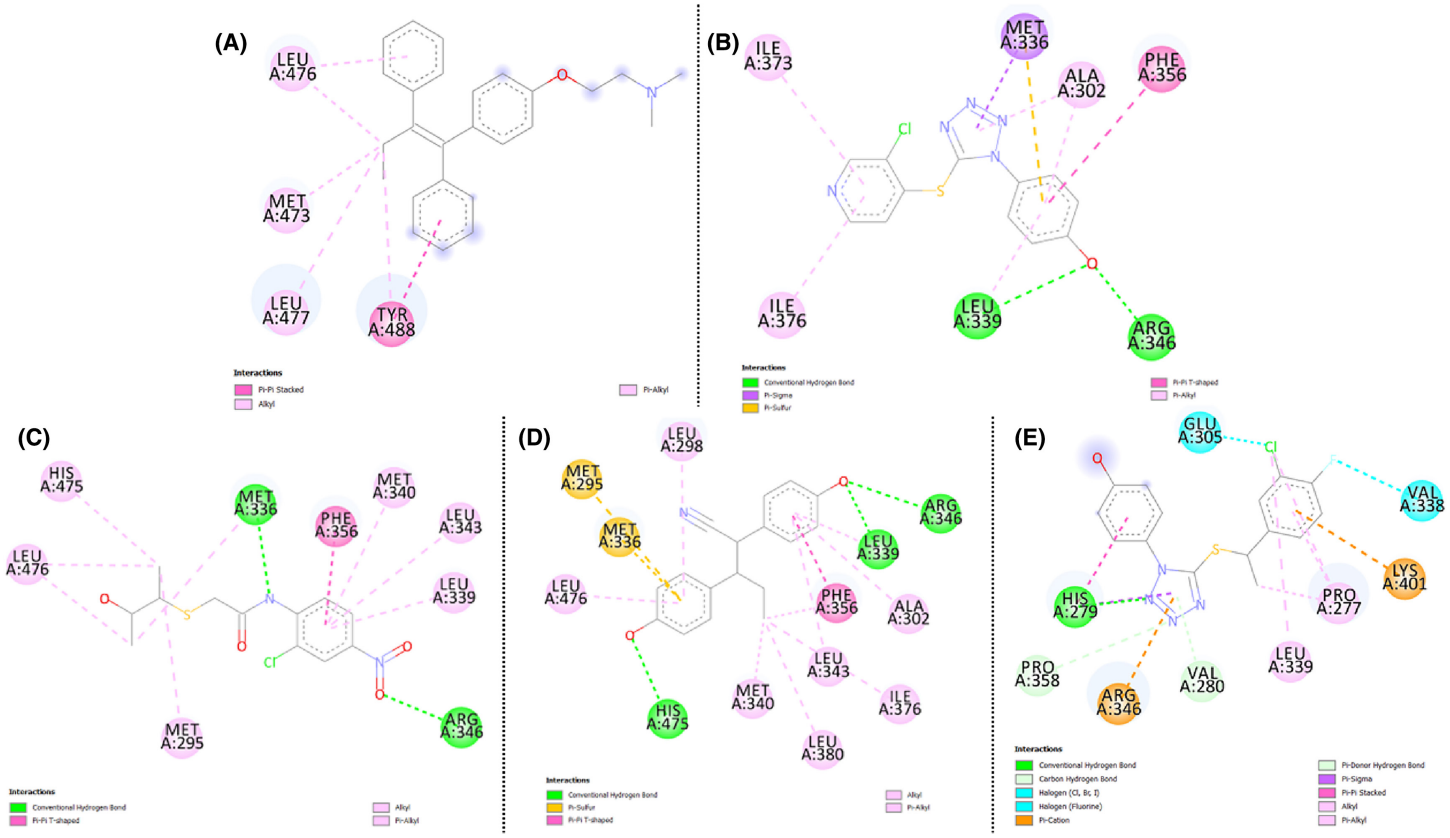
Molecular interaction of (A) Control (Tamoxifen), (B) ZINC94272748, (C) ZINC79046938, (D) ZINC05925939, and (E) ZINC59928516 with ESR2 protein 1QKM.

**FIGURE 10:**
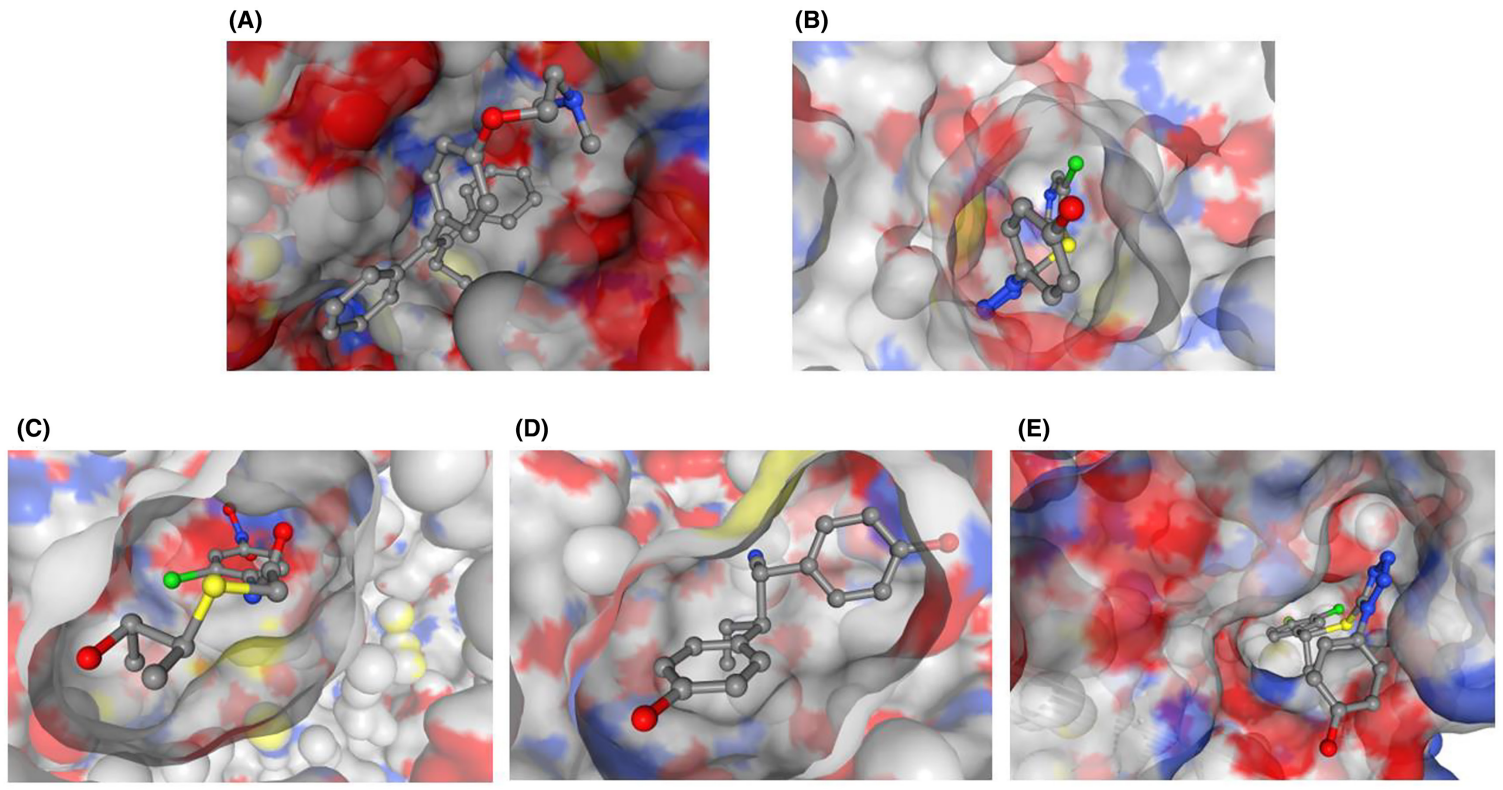
Molecular Interaction poses of (A) Control (Tamoxifen), (B) ZINC94272748, (C) ZINC79046938, (D) ZINC05925939, and (E) ZINC59928516 in the pocket of ESR2 protein 1QKM.

The molecular docking analysis of the top four compounds‐ ZINC94272748, ZINC79046938, ZINC05925939, and ZINC59928516 against the wild‐type ESR2 protein 1QKM has yielded insightful results, as summarized in Table 4. The compounds ZINC05925939 and ZINC59928516 exhibit the highest docking (XP Glide) Scores of −10.80 and −8.42 respectively, suggesting robust binding affinities that exhibits promising interactions with the wild‐type ESR2 protein. Figures 8, 9, 10 demonstrated the protein‐ligand docking complexes (3D), protein‐ligand interactions (2D), and docking pockets visualization respectively. These findings underscore their potential as lead compounds for further investigation in developing targeted therapies against breast cancer. The interacted amino acid residues are shown in Table S4.

**TABLE 4:** Docking results of top four hit compounds and a control drug (Tamoxifen).

Ligand	XP Glide Score	Cavity volume (Å^3^)	Center (x, y, z)	Docking size (x, y, z)
Control (Tamoxifen)	−7.2	238	15, 0, 113	23, 23, 23
ZINC94272748	−8.26	950	28, 11, 110	21, 21, 21
ZINC79046938	−5.73	950	28, 11, 110	21, 21, 21
ZINC05925939	−10.80	950	28, 11, 110	20, 20, 20
ZINC59928516	−8.42	950	28, 11, 110	22, 22, 22

### 3.7 Analysis of molecular dynamics simulations (MDS): RSMD, RMSF, RG, SASA, MolSA, PSA, and torsion angels

**FIGURE 11:**
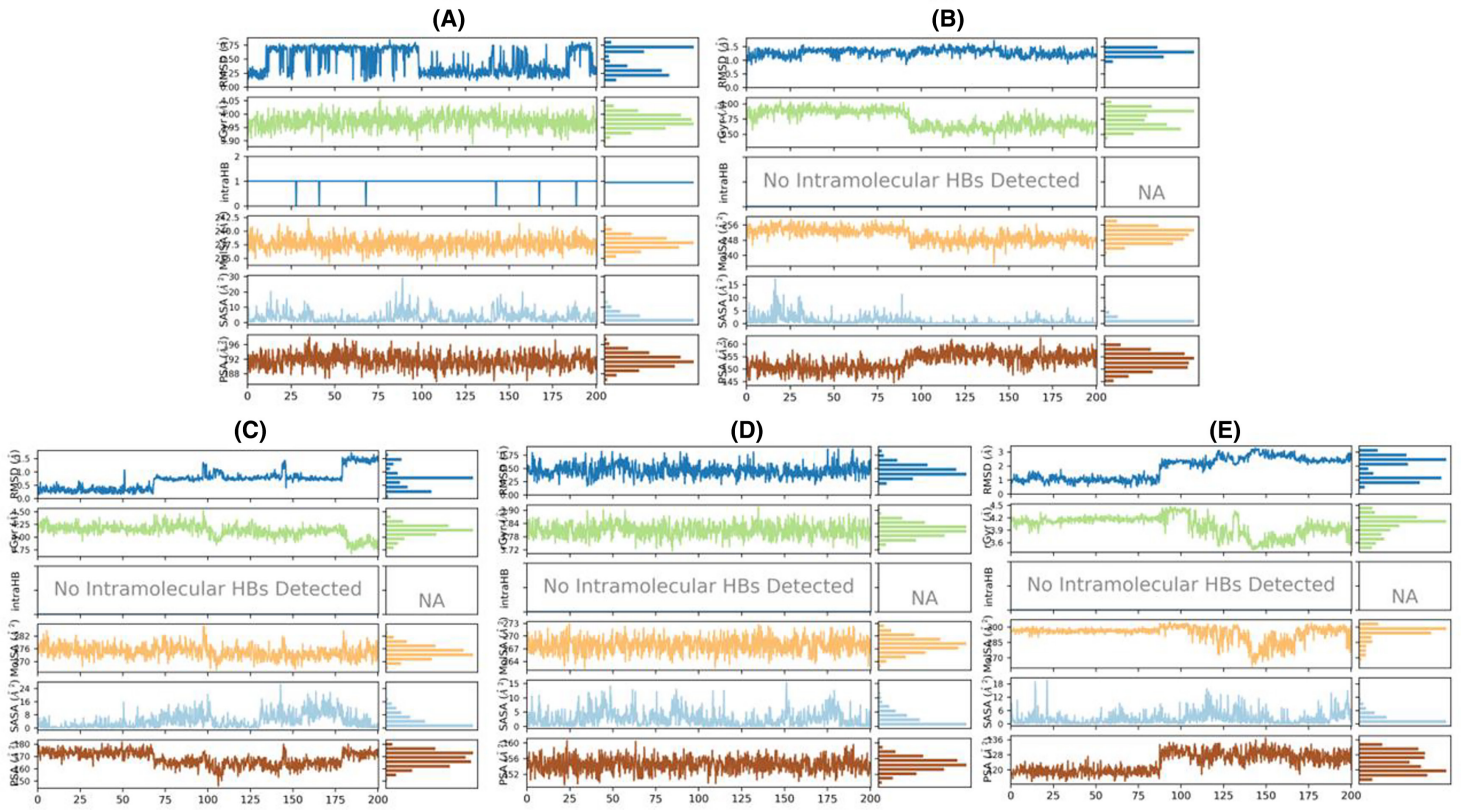
Molecular dynamics simulations: Properties (PSA, SASA, MolSA, IntraHB, rGyr, and RMSD) of the control and the top four compounds: (A) Control (Tamoxifen), (B) ZINC94272748, (C) ZINC79046938, (D) ZINC05925939, and (E) ZINC59928516.

In the comparative analysis of MD simulation results with the control (Tamoxifen), discernible distinctions were observed in the structural dynamics and surface properties of each compound (Table S5). Tamoxifen, serving as the control, exhibited notable structural stability, with a low RMSD ranging from 0 to 0.844 Å, indicative of a well‐preserved structure during the simulation. Furthermore, Tamoxifen displayed a consistent RMSF profile, with the highest fluctuation at atom no: 3 (1.984 Å) and the lowest at atom no: 14 (0.997 Å), resulting in an average RMSF of 1.484 Å (Figure 14A). The RG values for Tamoxifen ranged from 3.887 to 4.055 Å, emphasizing stable protein folding, while SASA values ranged from 0 to 29.341 Å^2^, with an average of 3.399 Å^2^, denoting the SASA (Figure 11A). Moving to the evaluated compounds, ZINC94272748 and ZINC79046938 demonstrated moderate structural stability, as reflected in their RMSD values ranging from 0 to 1.727 Å. These compounds exhibited variable RMSF profiles, suggesting fluctuating degrees of flexibility during the simulation (Figure 14B,C). The RG values for ZINC94272748 and ZINC79046938 indicated some variability in protein folding, emphasizing a dynamic conformational landscape. Additionally, SASA values ranging from 0 to 17.46 Å^2^ for ZINC94272748 and from 0 to 26.647 Å^2^ for ZINC79046938 illustrated diverse solvent accessibility (Figure 11B,C). Contrastingly, ZINC05925939 showcased remarkable stability, with an RMSD ranging from 0 to 0.872 Å, suggesting well‐maintained structural integrity throughout the simulation. The RMSF profile for ZINC05925939 exhibited the highest fluctuation at atom no: 18 (1.37 Å) and the lowest at atom no: 4 (0.636 Å), resulting in an average RMSF of 0.8984 Å (Figure 14D). The RG values indicated relatively stable protein folding, fluctuating between 3.714 and 3.914 Å. Furthermore, SASA values ranging from 0 to 15.508 Å^2^ reflected controlled solvent accessibility (Figure 11D). In the case of ZINC59928516, the compound displayed some structural deviation, as evidenced by an RMSD ranging from 0 to 3.268 Å. The RMSF profile indicated notable fluctuations, with the highest at atom no: 20 (3.27 Å) and the lowest at atom no: 9 (1.017 Å), resulting in an average RMSF of 1.88613 Å (Figure 14E). The RG values for ZINC59928516 suggested variability in protein folding, ranging from 3.426 to 4.483 Å. Additionally, SASA values ranging from 0 to 19.732 Å^2^ showcased diverse solvent accessibility (Figure 11E). Understanding the dynamic behavior of amino acids and identifying specific modification sites within a protein are crucial elements for interpreting functional dynamics during MD simulations. The root mean square fluctuation (RMSF) analysis provides valuable insights into the variability of individual amino acids over the simulation trajectory.

The average RMSF values for the ESR2 protein (1QKM) were determined as follows for the compounds: Control (Tamoxifen)—1.125066 Å, ZINC94272748–1.284402 Å, ZINC79046938–1.357149 Å, ZINC05925939–1.06692 Å, and ZINC59928516–1.06471 Å. Notable fluctuation was also observed in some residues, including PRO_285 (3.146 Å), SER_286 (2.9 Å), ALA_287 (2.681 Å), PRO_288 (2.232 Å), THR_417 (4.103 Å), ALA_416 (3.04 Å), THR_417 (2.412 Å), GLN_418 (2.533 Å), VAL_499 (2.645 Å), and LEU_500 (3.756 Å), particularly in the presence of the control compound Tamoxifen (Figure 13A). Furthermore, certain amino acid residues, such as HIS_279, VAL_280, LEU_281, ILE_282, SER_283, ARG_284, PRO_285, SER_286, ALA_287, PRO_288, PHE_289, THR_290, and GLU_291, exhibited higher RMSF values ranging from 2.5 to 4.5 Å for ZINC94272748 and 1.9–3.5 Å for the other compounds (Figure 13A–E). Notably, none of these highly fluctuating amino acid residues were directly involved in protein‐ligand contacts. Despite the observed fluctuations, the protein‐ligand complexes for all four compounds remained stable throughout the simulation. Notably, the protein 1QKM demonstrated enhanced stability, particularly in conjunction with ZINC05925939 and ZINC59928516 (Figure 13D,E).

**FIGURE 12:**
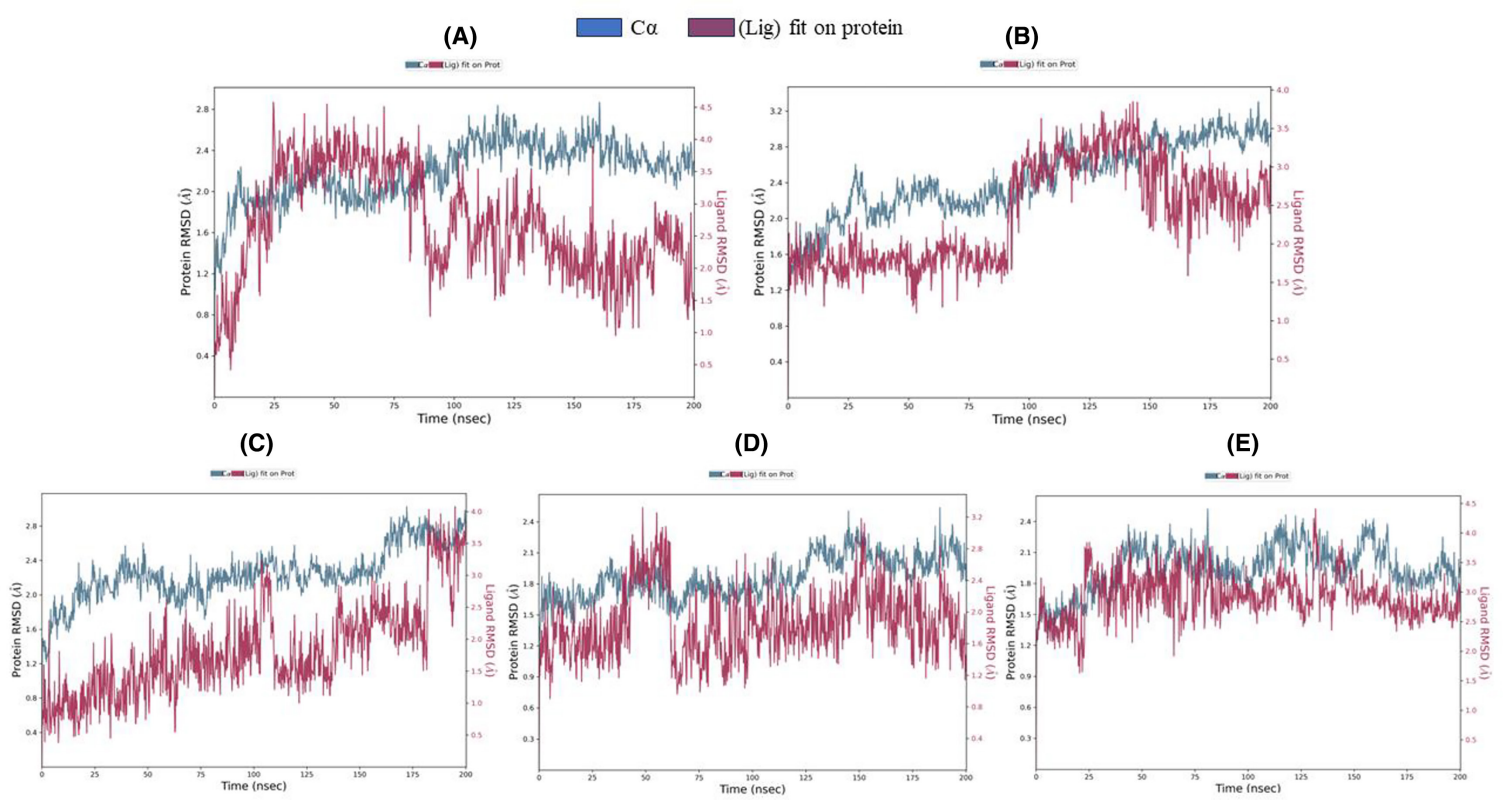
Root mean square deviation (RMSD) of 1QKM complex with (A) Control (Tamoxifen), (B) ZINC94272748, (C) ZINC79046938, (D) ZINC05925939, and (E) ZINC59928516.

A 2D schematic representation of the compounds is depicted, featuring color‐coded rotatable bonds (Figure S2). To enhance the visualization of rotatable torsional bonds, a dial (radial) plot, and corresponding color bar plots were incorporated. The radial plot illustrates the conformation of torsion angles throughout the simulation, originating from the center and progressing radially outward with time. Bar plots on the radial diagram summarize the probability density of torsion angles, expressing the data in kcal/mol on the y‐axis. Together, the radial and bar diagrams elucidate the relationships between torsion potential and conformational strain of the compounds while maintaining a protein‐bound conformation. Combining these MDS analyses of individual compounds, ZINC05925939 and ZINC59928516 remain noteworthy for their stable behavior both individually and within the protein‐ligand complex. The lower average RMSD of ZINC05925939 and ZINC59928516 (1.830682 and 1.943247 Å) in the protein (1QKM)‐ligand complex (Figure 12D,E) further support their potential, demonstrating a stability level even superior to the control (Tamoxifen). The summary of these results is presented in the Table 5.

**TABLE 5:** Molecular dynamics simulations: The Summary of properties of the control and the top four compounds.

Ligand	Protein‐ ligand complex	Ligand					
	RMSD (Å)	RMSD (Å)	RMSF (Å)	RG (Å)	SASA (Å^2^)	MolSA (Å^2^)	PSA (Å^2^)
Tamoxifen	2.181674	0.46445	1.48435	3.973084	3.39943	237.7174	191.56
ZINC94272748	2.413691	1.275028	1.63705	3.748928	1.058401	250.6109	153.1312
ZINC79046938	2.217276	0.699878	1.5681	4.122775	4.328577	274.5362	168.0278
ZINC05925939	1.830682	0.457712	0.8984	3.809431	2.668359	267.8755	154.4766
ZINC59928516	1.943247	1.843805	1.88613	4.013935	2.353914	294.0014	124.0335

**FIGURE 13:**
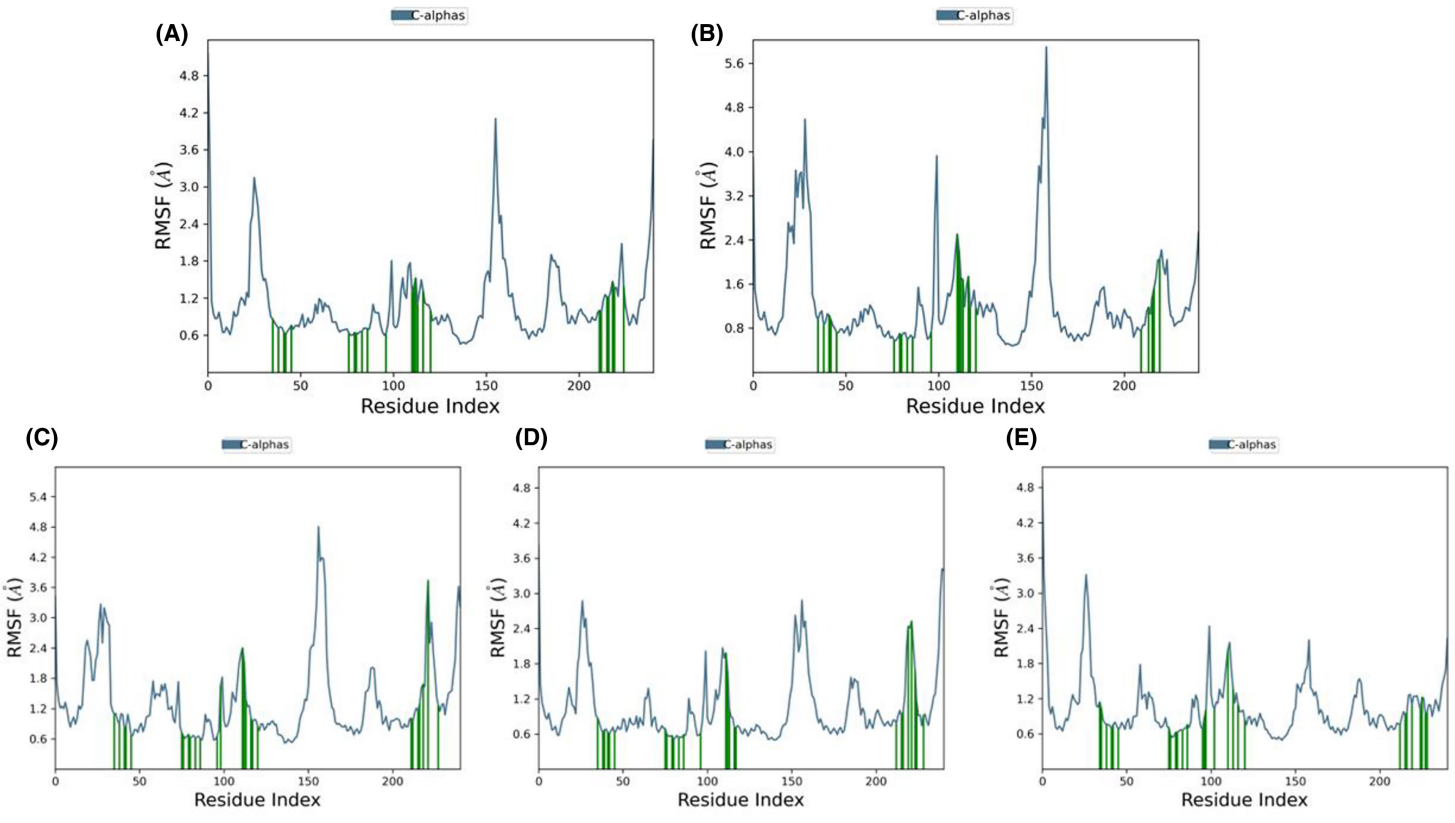
Molecular dynamics simulation: RMSF plotting of ESR2 protein 1QKM with (A) Control (Tamoxifen), (B) ZINC94272748, (C) ZINC79046938, (D) ZINC05925939, and (E) ZINC59928516. Green lines represent loop regions.

**FIGURE 14:**
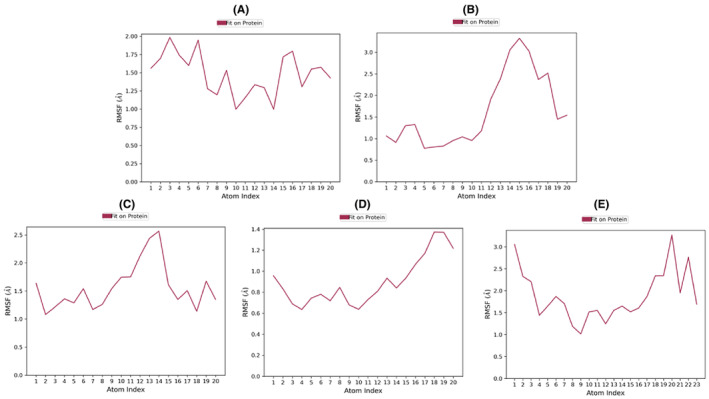
Root mean square fluctuation (RMSF) of (A) Control (Tamoxifen), (B) ZINC94272748, (C) ZINC79046938, (D) ZINC05925939, and (E) ZINC59928516.

**FIGURE 15:**
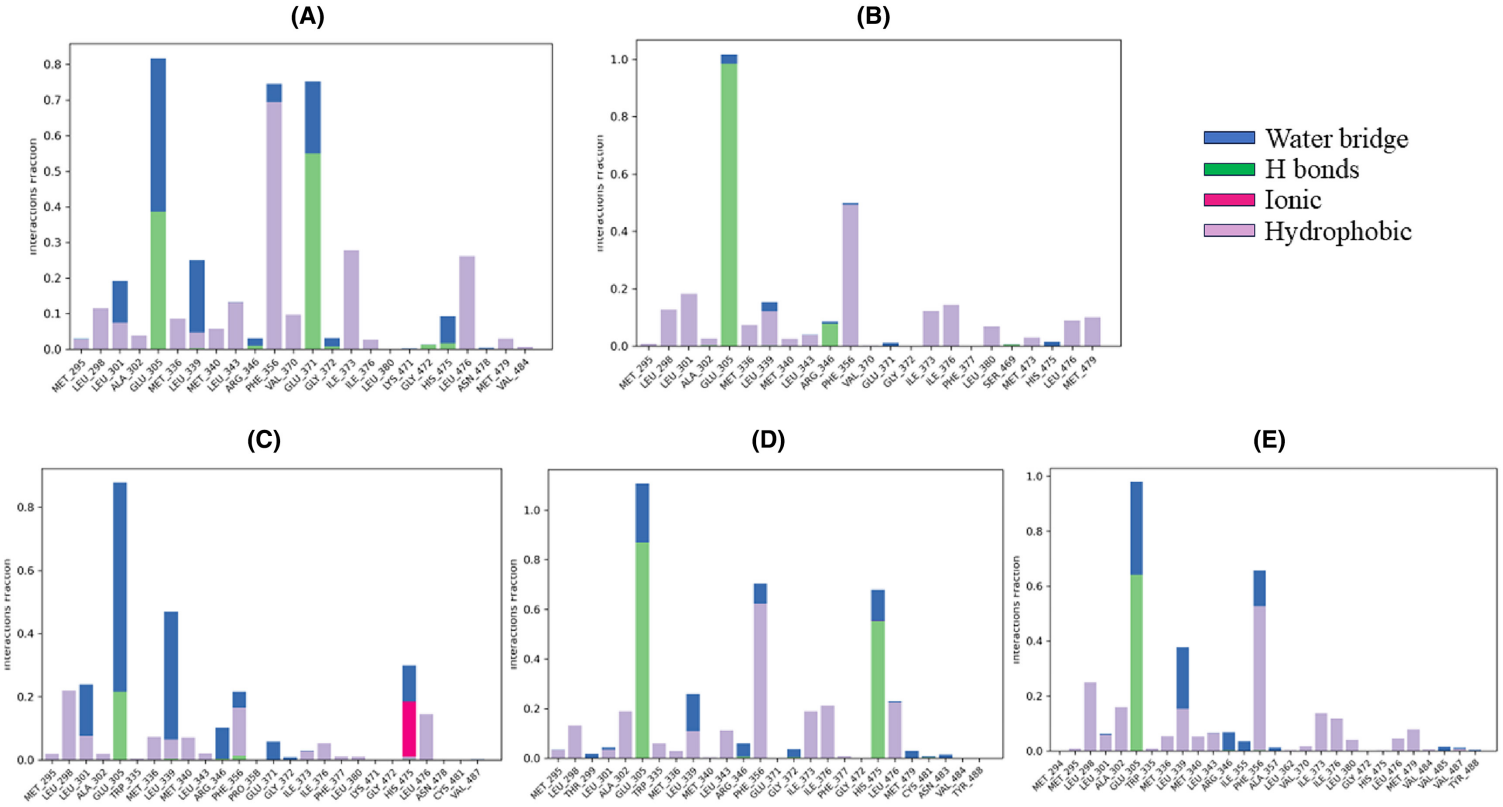
Plot (stacked bar charts) of the ligands (A) Control (Tamoxifen), (B) ZINC94272748, (C) ZINC79046938, (D) ZINC05925939, and (E) ZINC59928516 interactions with Protein (1QKM) supervised throughout the simulation period of 200 ns.

**FIGURE 16:**
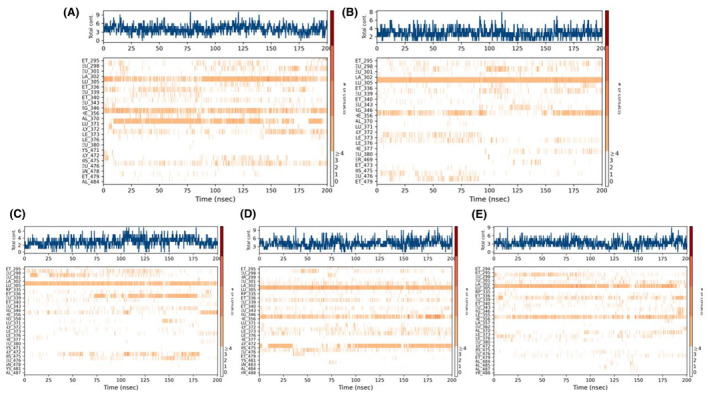
A timeline representation of the contacts (H‐bonds, hydrophobic, ionic and water bridges) of Protein (1QKM) interactions with the ligands (A) Control (Tamoxifen), (B) ZINC94272748, (C) ZINC79046938, (D) ZINC05925939, and (E) ZINC59928516 supervised throughout the simulation period of 200 ns. The top panel (in every figure) shows the total number of specific contacts the protein makes with the ligand over the course of the trajectory. The bottom panel (in every figure) shows which residues interact with the ligand in each trajectory frame. Some residues make more than one specific contact with the ligand, which is represented by a darker shade of orange, according to the scale to the right of the plot.

### 3.8 Analysis of MDS: protein‐ligand contacts

The top four compounds and control drug (Tamoxifen) were analyzed based on their interactions with the ESR2 protein (1QKM) during the 200 ns MD simulation, considering parameters such as hydrogen bonds, hydrophobic interactions, water bridges, and ionic interactions. (Figure 15A–E). In terms of hydrogen bond persistence, ZINC94272748 demonstrated the highest duration, with interactions lasting 99% of the simulation period (Figure 15B). This suggests a strong and stable binding affinity for ZINC94272748 compared to the other ligands. Control (Tamoxifen) also exhibited substantial hydrogen bond interactions, persisting for 38% of the simulation (Figure 15A). ZINC05925939 and ZINC59928516 showed intermediate levels of hydrogen bond persistence (84% and 63%, respectively), while ZINC79046938 displayed the lowest duration of 21% (Figure 15C–E). For hydrophobic interactions, ZINC94272748 again stood out, with a significant duration of 50%, indicating a robust and stable engagement with hydrophobic amino acid residues (Figure 15B). Control (Tamoxifen) and ZINC05925939 demonstrated substantial hydrophobic interactions, persisting for 70% and 63%, respectively (Figure 15A,D). ZINC59928516 displayed a notable duration of 56%, while ZINC79046938 exhibited a lower duration of 19% (Figure 15B,E). Concerning water bridges, ZINC05925939 formed the highest number of interactions, persisting with 12 amino acid residues for over 10% of the simulation (Figure 15D). ZINC79046938 displayed a substantial water bridge interaction duration of 65% (Figure 15E), further contributing to its binding stability. ZINC94272748 and ZINC59928516 formed water bridges with varying durations, with Control (Tamoxifen) exhibiting the lowest about 20% of simulation time (Figure 15A,E). Notably, ZINC79046938 was the only ligand to establish an ionic interaction, persisting for 20% of the simulation time (Figure 15C). This unique feature could have implications for its binding affinity and selectivity. A timeline of protein‐ligand contacts is presented graphically in Figure 16.

Considering the overall interaction profile, ZINC05925939 consistently demonstrated extended durations in both hydrogen bonds and hydrophobic interactions and water bridges, suggesting superior binding stability among the compounds. ZINC94272748 and Control (Tamoxifen) also displayed strong interaction profiles, while ZINC79046938 showed uniqueness with additional ionic interaction. In conclusion, based on the comprehensive analysis of protein‐ligand interactions, ZINC05925939 emerges as a promising candidate with the highest overall binding stability among the compounds studied.

### 3.9 MM‐GBSA analysis

The MM‐GBSA analysis provides insights into the binding free energy components for each ligand. ΔG Bind represents the total binding free energy, which is the sum of various contributions. Notably, ZINC05925939 exhibits the most negative ΔG Bind value (−55.15 kcal/mol), suggesting the highest overall binding affinity among the ligands. The Coulombic contribution (ΔG Coulomb) indicates electrostatic interactions, ZINC05925939 showing a significant contribution of −9.07 kcal/mol.^57^ The covalent contribution (ΔG Covalent) and hydrogen bonding contribution (ΔG H Bond) provide information on chemical and hydrogen bonding interactions, respectively. ZINC59928516 demonstrates a relatively high covalent contribution of 13.64 kcal/mol, while ZINC79046938 shows (8.03 kcal/mol) notable hydrogen bonding effects. Lipophilic interactions (ΔG Lipo) contribute favorably to binding in all ligands, with ZINC94272748 having the largest lipophilic contribution of −25.50 kcal/mol. The packing interactions (ΔG Packing)^58^ and self‐contact interactions (ΔG SelfCont) also contribute to binding. Solvation energies (ΔG Solv_GB and ΔG Solv_SA) indicate the ligands' interactions with the solvent; ZINC59928516 shows the highest solvation energy of 18.40 kcal/mol. Van der Waals interactions (ΔG vdW) play a crucial role, with ZINC05925939 exhibiting the most negative value is −41.84 kcal/mol. Overall, ZINC05925939 stands out as the ligand with the most favorable binding free energy, attributed to a combination of strong electrostatic, covalent, hydrogen bonding, lipophilic, and van der Waals interactions. The MM‐GBSA results are summarized in Table 6.

**TABLE 6:** MM‐GBSA analysis.

Ligands	ΔG Bind	ΔG Coulomb	ΔG Covalent	ΔG H Bond	ΔG Lipo	ΔG Packing	ΔG Solv_GB	ΔG vdW	ΔG SelfCont	ΔG Solv_SA	Lig_Energy
ZINC94272748	−49.39	−10.97	8.47	−1.00	−25.50	−0.53	16.26	−36.12	0	‐	16.30
ZINC79046938	−48.12	−10.72	8.03	−2.07	−25.39	−5.39E‐05	16.00	−33.98	0	‐	9.98
ZINC05925939	−55.15	−9.07	9.03	−1.50	−27.70	−0.66	16.60	−41.84	0	‐	11.29
ZINC59928516	−40.79	−12.01	13.64	−1.01	−32.70	−0.61	18.40	−26.49	0	‐	23.15

*Note*: Here, ΔG Bind: The total binding free energy calculated by MMGBSA. This is the sum of various energy contributions. ΔG Bind Coulomb: Coulombic contribution to the binding free energy. ΔG Bind Covalent: contribution to the binding free energy. ΔG Bind Hbond: Contribution from hydrogen bonding to the binding free energy. ΔG Bind Lipo: Contribution from lipophilic interac221tions to the binding free energy. ΔG Bind Packing: Contribution from packing interactions to the binding free energy. ΔG Bind SelfCont: Contribution from self‐contact interactions to the binding free energy. ΔG Bind GB: Contribution from the generalized Born solvation energy to the binding free energy. ΔG Bind Solv_SA: Contribution from the solvent accessible surface area (SA) to the binding free energy. ΔG Bind vdW: van der Waals contribution to the binding free energy. Strain_Energy: Energy associated with ligand strain.

### 3.10 ADMET analysis

The ADMET analysis provides crucial information on the absorption, distribution, metabolism, excretion, and toxicity of the compounds, aiding in the assessment of their pharmacokinetic properties. Tamoxifen, used as a control, exhibits moderate absorption (C2P: 1.065) and high human intestinal absorption (HIA: 96.885%). It is a P‐glycoprotein inhibitor (P‐gpI), indicating potential interactions with this transporter. Tamoxifen demonstrates blood–brain barrier (BBB) permeability (log BB: 1.329) and central nervous system (CNS) permeability (log PS: −1.473), suggesting its ability to enter the brain. Among the studied compounds, ZINC94272748 shows moderate absorption (C2P: 1.264) and high HIA (93.834%), but it is not a P‐gpI. It exhibits limited BBB permeability (log BB: −0.994) and low CNS permeability (log PS: −2.525), indicating reduced potential for central nervous system effects. ZINC79046938 has lower absorption (C2P: 0.812) and HIA (77.077%) but is a P‐gpI, suggesting potential efflux interactions. It shows BBB permeability (log BB: −0.621) and limited CNS permeability (log PS: −2.55). ZINC05925939 exhibits moderate absorption (C2P: 1.095) and high HIA (91.493%), with BBB permeability (log BB: −0.39) and moderate CNS permeability (log PS: −1.908). ZINC59928516 has high absorption (C2P: 1.244) and HIA (91.854%) but is not a P‐gpI. It shows limited BBB permeability (log BB: −0.083) and low CNS permeability (log PS: −2.246).

In terms of toxicity, all compounds show no hepatotoxicity (HT = No). ZINC94272748 and ZINC05925939 exhibit potential for hERG inhibition,^51^ suggesting a possibility of cardiac effects. Tamoxifen and ZINC59928516 have LD50 values indicating moderate acute oral toxicity in rats, while the others fall within a similar range. Overall, these ADMET predictions highlight variations in the pharmacokinetic properties and potential toxicities of the studied compounds, providing valuable information for further drug development considerations. The results of the ADMET analysis are summarized in Table 7.

**TABLE 7:** ADMET analysis of the top four compounds.

Compounds	Absorption			Distribution		Metabolism	Excretion	Toxicity		
	C2P	HIA (%)	P‐gpI	BBB	CNS	CYP1A2	TC	hERGI	LD_50_ (rat)	HT
				(Permeability)						
Tamoxifen (control)	1.065	96.885	Yes	1.329	−1.473	Yes	0.556	Yes	2.285	No
ZINC94272748	1.264	93.834	No	−0.994	−2.525	No	−0.099	No	2.618	No
ZINC79046938	0.812	77.077	Yes	−0.621	−2.55	Yes	0.614	No	2.317	No
ZINC05925939	1.095	91.493	Yes	−0.39	−1.908	Yes	0.324	Yes	2.167	No
ZINC59928516	1.244	91.854	No	−0.083	−2.246	No	−0.16	No	2.669	Yes

*Note*: Here, TC = total clearance and measured in log mL/min/kg; I = inhibitor; Caco‐2 permeability (C2P) (log Papp in 10^−6^ cm/s); human intestinal absorption (HIA) (% absorbed), and P‐glycoprotein inhibitor (P‐gpI); The blood–brain barrier (BBB) (log BB) and central nervous system (CNS) permeability (log PS); human ether‐a‐go‐go‐gene (hERG) and oral rat acute toxicity (LD50) (mol/kg); HT = Hepatotoxicity.

## 4 DISCUSSION

The exploration of ESR2 mutations and subsequent drug discovery efforts involve a detailed analysis of both structural and pharmacokinetic aspects. We initiated our investigation by scrutinizing the structural disparities between the wild‐type ESR2 (PDB ID 1QKM) and its mutated counterparts (PDB IDs 2FSZ, 7XVZ, and 7XWR). Notably, these mutations introduced variations in the ligand‐binding domain, marked by amino acid substitutions at specific positions, such as C78S, C113S, and C225S. This structural divergence formed the groundwork for our pharmacophoric modeling using LigandScout. The generated pharmacophore models, representing key features like HBA, Hydrogen bond donor (HBD), and Ar, were aligned to produce a SFP. This SFP, a consolidation of essential molecular features crucial for potential drug interactions, served as a template for virtual screening.

In the subsequent steps, we meticulously created a ligand library, conducted virtual screening, and applied the Lipinski Rule of Five for stringent filtering. The top four compounds that emerged from this process—ZINC94272748, ZINC79046938, ZINC05925939, and ZINC59928516—were subjected to an in‐depth analysis. Analyzing the physicochemical properties, we observed that these compounds exhibited drug‐like characteristics, surpassing Lipinski,^59^ Ghose,^60^ Veber,^61^ Egan,^62^ and Muegge^63^ criteria. Notably, ZINC94272748 displayed a lower lipophilicity (Consensus Log Po/w) compared to other ligands.

Molecular docking analysis against the wild‐type ESR2 protein (PDB ID: 1QKM) unveiled ZINC05925939 and ZINC59928516 as top hits, demonstrating robust binding affinities. To delve deeper into their behavior, we conducted MD simulations, revealing stability patterns. ZINC05925939 showcased remarkable stability, suggesting its potential as a lead compound for breast cancer therapeutics. This observation was further corroborated by its lower average RMSD in the protein‐ligand complex compared to the control (Tamoxifen). Moving to the MM‐GBSA analysis, ZINC05925939 exhibited the most negative ΔG Bind value, signifying the highest overall binding affinity among the ligands. The diverse contributions to the binding free energy, including electrostatic, covalent, hydrogen bonding, lipophilic, and van der Waals interactions, underscored the multifaceted nature of its binding mechanism. In the realm of ADMET analysis, all compounds demonstrated no hepatotoxicity. ZINC94272748 and ZINC05925939 displayed potential for hERG inhibition, suggesting possible cardiac effects. Notably, the predicted LD50 values indicated moderate acute oral toxicity in rats for Tamoxifen and ZINC59928516.

In recent years, advancements in computer‐aided drug design (CADD) have shown promising potential in optimizing and screening molecules for in vitro and in vivo studies, although realistic applications remain limited due to unresolved challenges.^64^ Similarly, pharmacophore studies have certain limitations that can be improved. Utilizing computational programs with high accuracy could reduce these limitations and streamline the drug design process.^65^ The integration of machine learning (ML) approaches, such as deep learning, with extensive biological data from databases has provided significant insights into chemical structures, aiding in the prediction of clinical outcomes and advancing drug discovery. For example, virtual screening of flavonoids has identified potential novel inhibitors for targets like HDAC2 and VEGFR2.^66^, ^67^ These studies highlight the potential natural compounds, such as soybean‐derived isoflavone genistein, in targeting breast cancer signaling proteins, offering new avenues for nutraceutical development.^68^ Similarly, investigations into estrogen receptor beta (ERβ, encoded by ESR2) in breast cancer have revealed its association with improved overall survival and immune response modulation, suggesting that high ESR2 expression, particularly in basal‐ and normal‐like subtypes, may correlate with favorable prognosis.^2^ These findings underscore the significance of integrating in‐silico methods and biological data to identify and validate potential drug candidates, aligning with the aim of our research to target mutant ESR2 proteins using pharmacophore modeling and drug database screening.

In conclusion, our integrated approach, spanning structural analysis, pharmacophore modeling, virtual screening, physicochemical property evaluation, molecular docking, and ADMET analysis, has identified ZINC05925939 as a promising candidate for further investigation in breast cancer therapy. This compound exhibited favorable characteristics across multiple facets of drug development, highlighting the efficacy of our comprehensive computational methodology. Experimental validation will be imperative to solidify the therapeutic potential of these identified compounds.

## 5 CONCLUSION

In conclusion, our comprehensive computational study focused on understanding the structural implications of ESR2 mutations and exploring potential drug candidates for breast cancer therapy. Through a meticulous analysis of the wild‐type and mutated ESR2 proteins, pharmacophore modeling, virtual screening, and in‐depth evaluations of physicochemical, molecular docking, dynamics, MM‐GBSA, and ADMET properties, we have identified ZINC05925939 as a standout candidate. ZINC05925939 exhibited a remarkable binding affinity, reflected in its negative ΔG Bind value and superior stability during MD simulations. The shared pharmacophoric features, alongside favorable physicochemical properties and absence of hepatotoxicity, position ZINC05925939 as a promising lead compound. While other candidates, such as ZINC94272748, ZINC79046938, and ZINC59928516, also demonstrated notable attributes, the multifaceted strengths of ZINC05925939 make it particularly compelling for further experimental validation. This study not only sheds light on potential therapeutic candidates for breast cancer but also underscores the efficacy of our integrative computational approach in drug discovery. Moving forward, experimental investigations will be crucial to validate and refine these findings, paving the way for the development of targeted and effective breast cancer interventions.

## AUTHOR CONTRIBUTIONS

**Sirajul Islam:** Conceptualization (equal); data curation (equal); formal analysis (lead); methodology (equal); validation (equal); writing – original draft (lead). **Md. Al Amin:** Data curation (equal); formal analysis (equal); investigation (equal); methodology (equal); validation (equal); writing – original draft (supporting). **Kannan R. R. Rengasamy:** Data curation (equal); formal analysis (equal); methodology (equal); validation (equal). **A. K. M. Mohiuddin:** Formal analysis (equal); investigation (equal); methodology (equal); supervision (supporting); validation (equal); writing – review and editing (supporting). **Shahin Mahmud:** Conceptualization (equal); data curation (equal); formal analysis (equal); investigation (lead); methodology (lead); supervision (lead); validation (lead); writing – review and editing (lead).

## CONFLICT OF INTEREST STATEMENT

None.

## Supporting information


Figure S1.

Table S1.

## Data Availability

All data included in the study will be available for everyone as per journal policy.
