# Correction to “Structure‐based pharmacophore modeling for precision inhibition of mutant ESR2 in breast cancer: A systematic computational approach”

**DOI:** 10.1002/cam4.70174

**Published:** 2024-09-09

Islam S, Amin MA, Rengasamy KRR, Mohiuddin AKM, Mahmud S. Structure‐based pharmacophore modeling for precision inhibition of mutant ESR2 in breast cancer: A systematic computational approach. *Cancer Med*. 2024;13(15):e70074. doi:10.1002/cam4.70074.

A second affiliation has been added for Kannan R. R. Rengasamy and reads as follows:

^3^Centre of Excellence for Pharmaceutical Sciences, North‐West University, Potchefstroom, 2520, South Africa.

An acknowledgment section has been added and reads as follows:

Kannan R.R. Rengasamy acknowledges the Centre for High Performance Computing (CHPC), South Africa, for providing computational resources to this research project.

We apologize for this error.

