# Supplementary material for: Structure‐based pharmacophore modeling for precision inhibition of mutant ESR2 in breast cancer: A systematic computational approach
Source: Cancer Med. 2024 Aug 5;13(15):e70074. doi: 10.1002/cam4.70074 (PMC11299079; doi:10.1002/cam4.70074)
Supplement: Supplementary file 1 — Figure S1. [file CAM4-13-e70074-s001.docx]

**Supplementary Figures:**

**
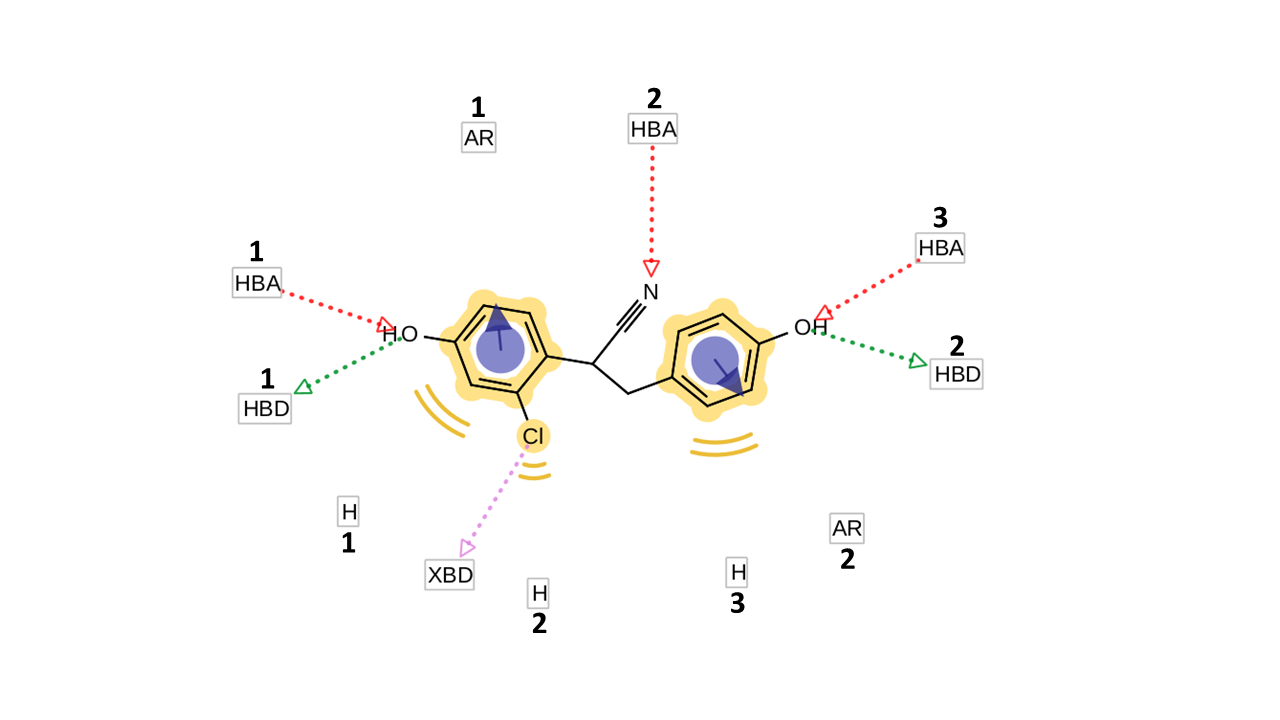
**

**Figure S1:** Pharmacophoric Features of SFP Model.


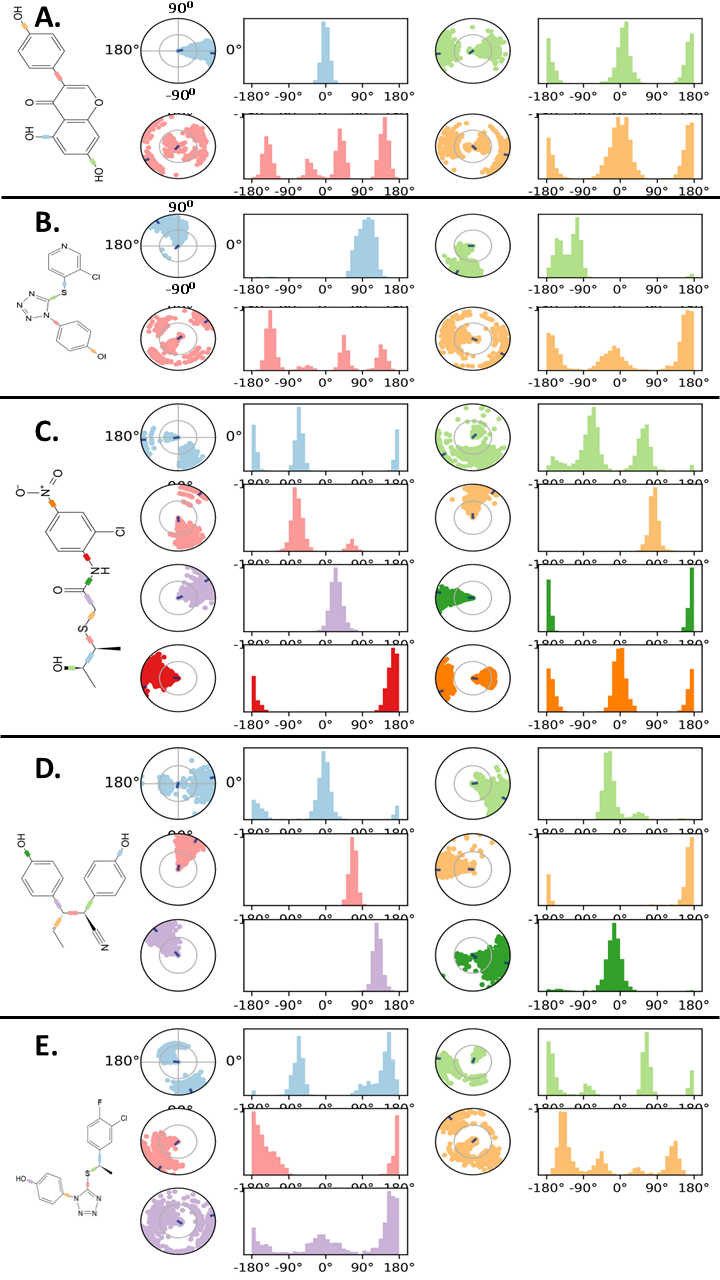


**Figure S2:** Torsions of **(A)** Control (Tamoxifen), **(B)** ZINC94272748, **(C)** ZINC79046938, **(D)** ZINC05925939 and **(E)** ZINC59928516.
