# Supplementary material for: Structure‐based pharmacophore modeling for precision inhibition of mutant ESR2 in breast cancer: A systematic computational approach
Source: Cancer Med. 2024 Aug 5;13(15):e70074. doi: 10.1002/cam4.70074 (PMC11299079; doi:10.1002/cam4.70074)
Supplement: Supplementary file 2 — Table S1. [file CAM4-13-e70074-s002.docx]

**Table S1: 336 combinations of shared features pharmacophore (SFP Model) and resulted 41,248 hits after repeated 1^st^ round of Virtual Screening From 21.777093 million compounds of ZINCPharmer database.**

| **SL** | **Combinations** | **ZincPharmar Hits** |
| --- | --- | --- |
| 1 | Ar1, HBA1, HBD1, H1, H2 | 230 |
| 2 | Ar1, HBA1, HBD1, H1, H3 | 323 |
| 3 | Ar1, HBA1, HBD1, H1, XBD | 224 |
| 4 | Ar1, HBA1, HBD1, H1, Ar2 | 156 |
| 5 | Ar1, HBA1, HBD1, H1, HBA2 | 55 |
| 6 | Ar1, HBA1, HBD1, H1, HBA3 | 190 |
| 7 | Ar1, HBA1, HBD1, H1, HBD2 | 183 |
| 8 | Ar1, HBA1, HBD1, H2, H3 | 160 |
| 9 | Ar1, HBA1, HBD1, H2, XBD | 909 |
| 10 | Ar1, HBA1, HBD1, H2, Ar2 | 153 |
| 11 | Ar1, HBA1, HBD1, H2, HBA2 | 178 |
| 12 | Ar1, HBA1, HBD1, H2, HBA3 | 87 |
| 13 | Ar1, HBA1, HBD1, H2, HBD2 | 125 |
| 14 | Ar1, HBA1, HBD1, H3, XBD | 333 |
| 15 | Ar1, HBA1, HBD1, H3, Ar2 | 107 |
| 16 | Ar1, HBA1, HBD1, H3, HBA2 | 188 |
| 17 | Ar1, HBA1, HBD1, H3, HBA3 | 97 |
| 18 | Ar1, HBA1, HBD1, H3, HBD2 | 5 |
| 19 | Ar1, HBA1, HBD1, XBD, Ar2 | 34 |
| 20 | Ar1, HBA1, HBD1, XBD, HBA2 | 83 |
| 21 | Ar1, HBA1, HBD1, XBD, HBA3 | 3 |
| 22 | Ar1, HBA1, HBD1, XBD, HBD2 | 54 |
| 23 | Ar1, HBA1, HBD1, Ar2, HBA2 | 162 |
| 24 | Ar1, HBA1, HBD1, Ar2, HBA3 | 108 |
| 25 | Ar1, HBA1, HBD1, Ar2, HBD2 | 187 |
| 26 | Ar1, HBA1, HBD1, HBA2, HBA3 | 38 |
| 27 | Ar1, HBA1, HBD1, HBA2, HBD2 | 11 |
| 28 | Ar1, HBA1, HBD1, HBA3, HBD2 | 171 |
| 29 | Ar1, HBA1, HBD2, H1, H2 | 108 |
| 30 | Ar1, HBA1, HBD2, H1, H3 | 82 |
| 31 | Ar1, HBA1, HBD2, H1, XBD | 126 |
| 32 | Ar1, HBA1, HBD2, H1, Ar2 | 194 |
| 33 | Ar1, HBA1, HBD2, H1, HBA2 | 330 |
| 34 | Ar1, HBA1, HBD2, H1, HBA3 | 90 |
| 35 | Ar1, HBA1, HBD2, H1, HBD1 | 115 |
| 36 | Ar1, HBA1, HBD2, H2, H3 | 51 |
| 37 | Ar1, HBA1, HBD2, H2, XBD | 780 |
| 38 | Ar1, HBA1, HBD2, H2, Ar2 | 78 |
| 39 | Ar1, HBA1, HBD2, H2, HBA2 | 130 |
| 40 | Ar1, HBA1, HBD2, H2, HBA3 | 10 |
| 41 | Ar1, HBA1, HBD2, H2, HBD1 | 79 |
| 42 | Ar1, HBA1, HBD2, H3, XBD | 70 |
| 43 | Ar1, HBA1, HBD2, H3, Ar2 | 92 |
| 44 | Ar1, HBA1, HBD2, H3, HBA2 | 39 |
| 45 | Ar1, HBA1, HBD2, H3, HBA3 | 142 |
| 46 | Ar1, HBA1, HBD2, H3, HBD1 | 71 |
| 47 | Ar1, HBA1, HBD2, XBD, Ar2 | 144 |
| 48 | Ar1, HBA1, HBD2, XBD, HBA2 | 143 |
| 49 | Ar1, HBA1, HBD2, XBD, HBA3 | 12 |
| 50 | Ar1, HBA1, HBD2, XBD, HBD1 | 166 |
| 51 | Ar1, HBA1, HBD2, Ar2, HBA2 | 96 |
| 52 | Ar1, HBA1, HBD2, Ar2, HBA3 | 53 |
| 53 | Ar1, HBA1, HBD2, Ar2, HBD1 | 10 |
| 54 | Ar1, HBA1, HBD2, HBA2, HBA3 | 88 |
| 55 | Ar1, HBA1, HBD2, HBA2, HBD1 | 178 |
| 56 | Ar1, HBA1, HBD2, HBA3, HBD1 | 51 |
| 57 | Ar1, HBA2, HBD1, H1, H2 | 145 |
| 58 | Ar1, HBA2, HBD1, H1, H3 | 71 |
| 59 | Ar1, HBA2, HBD1, H1, XBD | 98 |
| 60 | Ar1, HBA2, HBD1, H1, Ar2 | 110 |
| 61 | Ar1, HBA2, HBD1, H1, HBA1 | 60 |
| 62 | Ar1, HBA2, HBD1, H1, HBA3 | 84 |
| 63 | Ar1, HBA2, HBD1, H1, HBD2 | 190 |
| 64 | Ar1, HBA2, HBD1, H2, H3 | 57 |
| 65 | Ar1, HBA2, HBD1, H2, XBD | 147 |
| 66 | Ar1, HBA2, HBD1, H2, Ar2 | 191 |
| 67 | Ar1, HBA2, HBD1, H2, HBA1 | 126 |
| 68 | Ar1, HBA2, HBD1, H2, HBA3 | 124 |
| 69 | Ar1, HBA2, HBD1, H2, HBD2 | 133 |
| 70 | Ar1, HBA2, HBD1, H3, XBD | 14 |
| 71 | Ar1, HBA2, HBD1, H3, Ar2 | 97 |
| 72 | Ar1, HBA2, HBD1, H3, HBA1 | 148 |
| 73 | Ar1, HBA2, HBD1, H3, HBA3 | 70 |
| 74 | Ar1, HBA2, HBD1, H3, HBD2 | 39 |
| 75 | Ar1, HBA2, HBD1, XBD, Ar2 | 53 |
| 76 | Ar1, HBA2, HBD1, XBD, HBA1 | 121 |
| 77 | Ar1, HBA2, HBD1, XBD, HBA3 | 85 |
| 78 | Ar1, HBA2, HBD1, XBD, HBD2 | 87 |
| 79 | Ar1, HBA2, HBD1, Ar2, HBA1 | 44 |
| 80 | Ar1, HBA2, HBD1, Ar2, HBA3 | 198 |
| 81 | Ar1, HBA2, HBD1, Ar2, HBD2 | 18 |
| 82 | Ar1, HBA2, HBD1, HBA1, HBA3 | 127 |
| 83 | Ar1, HBA2, HBD1, HBA1, HBD2 | 129 |
| 84 | Ar1, HBA2, HBD1, HBA3, HBD2 | 66 |
| 85 | Ar1, HBA2, HBD2, H1, H2 | 189 |
| 86 | Ar1, HBA2, HBD2, H1, H3 | 75 |
| 87 | Ar1, HBA2, HBD2, H1, XBD | 86 |
| 88 | Ar1, HBA2, HBD2, H1, Ar2 | 37 |
| 89 | Ar1, HBA2, HBD2, H1, HBA1 | 49 |
| 90 | Ar1, HBA2, HBD2, H1, HBA3 | 220 |
| 91 | Ar1, HBA2, HBD2, H1, HBD1 | 153 |
| 92 | Ar1, HBA2, HBD2, H2, H3 | 159 |
| 93 | Ar1, HBA2, HBD2, H2, XBD | 82 |
| 94 | Ar1, HBA2, HBD2, H2, Ar2 | 63 |
| 95 | Ar1, HBA2, HBD2, H2, HBA1 | 150 |
| 96 | Ar1, HBA2, HBD2, H2, HBA3 | 72 |
| 97 | Ar1, HBA2, HBD2, H2, HBD1 | 62 |
| 98 | Ar1, HBA2, HBD2, H3, XBD | 178 |
| 99 | Ar1, HBA2, HBD2, H3, Ar2 | 4 |
| 100 | Ar1, HBA2, HBD2, H3, HBA1 | 85 |
| 101 | Ar1, HBA2, HBD2, H3, HBA3 | 55 |
| 102 | Ar1, HBA2, HBD2, H3, HBD1 | 132 |
| 103 | Ar1, HBA2, HBD2, XBD, Ar2 | 66 |
| 104 | Ar1, HBA2, HBD2, XBD, HBA1 | 41 |
| 105 | Ar1, HBA2, HBD2, XBD, HBA3 | 99 |
| 106 | Ar1, HBA2, HBD2, XBD, HBD1 | 193 |
| 107 | Ar1, HBA2, HBD2, Ar2, HBA1 | 135 |
| 108 | Ar1, HBA2, HBD2, Ar2, HBA3 | 38 |
| 109 | Ar1, HBA2, HBD2, Ar2, HBD1 | 19 |
| 110 | Ar1, HBA2, HBD2, HBA1, HBA3 | 560 |
| 111 | Ar1, HBA2, HBD2, HBA1, HBD1 | 480 |
| 112 | Ar1, HBA2, HBD2, HBA3, HBD1 | 99 |
| 113 | Ar1, HBA3, HBD1, H1, H2 | 92 |
| 114 | Ar1, HBA3, HBD1, H1, H3 | 142 |
| 115 | Ar1, HBA3, HBD1, H1, XBD | 53 |
| 116 | Ar1, HBA3, HBD1, H1, Ar2 | 179 |
| 117 | Ar1, HBA3, HBD1, H1, HBA1 | 13 |
| 118 | Ar1, HBA3, HBD1, H1, HBA2 | 131 |
| 119 | Ar1, HBA3, HBD1, H1, HBD2 | 83 |
| 120 | Ar1, HBA3, HBD1, H2, H3 | 25 |
| 121 | Ar1, HBA3, HBD1, H2, XBD | 109 |
| 122 | Ar1, HBA3, HBD1, H2, Ar2 | 74 |
| 123 | Ar1, HBA3, HBD1, H2, HBA1 | 440 |
| 124 | Ar1, HBA3, HBD1, H2, HBA2 | 95 |
| 125 | Ar1, HBA3, HBD1, H2, HBD2 | 48 |
| 126 | Ar1, HBA3, HBD1, H3, XBD | 91 |
| 127 | Ar1, HBA3, HBD1, H3, Ar2 | 91 |
| 128 | Ar1, HBA3, HBD1, H3, HBA1 | 78 |
| 129 | Ar1, HBA3, HBD1, H3, HBA2 | 28 |
| 130 | Ar1, HBA3, HBD1, H3, HBD2 | 2 |
| 131 | Ar1, HBA3, HBD1, XBD, Ar2 | 111 |
| 132 | Ar1, HBA3, HBD1, XBD, HBA1 | 179 |
| 133 | Ar1, HBA3, HBD1, XBD, HBA2 | 95 |
| 134 | Ar1, HBA3, HBD1, XBD, HBD2 | 114 |
| 135 | Ar1, HBA3, HBD1, Ar2, HBA1 | 180 |
| 136 | Ar1, HBA3, HBD1, Ar2, HBA2 | 70 |
| 137 | Ar1, HBA3, HBD1, Ar2, HBD2 | 63 |
| 138 | Ar1, HBA3, HBD1, HBA1, HBA2 | 102 |
| 139 | Ar1, HBA3, HBD1, HBA1, HBD2 | 68 |
| 140 | Ar1, HBA3, HBD1, HBA2, HBD2 | 58 |
| 141 | Ar1, HBA3, HBD2, H1, H2 | 11 |
| 142 | Ar1, HBA3, HBD2, H1, H3 | 94 |
| 143 | Ar1, HBA3, HBD2, H1, XBD | 166 |
| 144 | Ar1, HBA3, HBD2, H1, Ar2 | 96 |
| 145 | Ar1, HBA3, HBD2, H1, HBA1 | 132 |
| 146 | Ar1, HBA3, HBD2, H1, HBA2 | 34 |
| 147 | Ar1, HBA3, HBD2, H1, HBD1 | 65 |
| 148 | Ar1, HBA3, HBD2, H2, H3 | 200 |
| 149 | Ar1, HBA3, HBD2, H2, XBD | 156 |
| 150 | Ar1, HBA3, HBD2, H2, Ar2 | 16 |
| 151 | Ar1, HBA3, HBD2, H2, HBA1 | 121 |
| 152 | Ar1, HBA3, HBD2, H2, HBA2 | 229 |
| 153 | Ar1, HBA3, HBD2, H2, HBD1 | 909 |
| 154 | Ar1, HBA3, HBD2, H3, XBD | 770 |
| 155 | Ar1, HBA3, HBD2, H3, Ar2 | 102 |
| 156 | Ar1, HBA3, HBD2, H3, HBA1 | 44 |
| 157 | Ar1, HBA3, HBD2, H3, HBA2 | 180 |
| 158 | Ar1, HBA3, HBD2, H3, HBD1 | 220 |
| 159 | Ar1, HBA3, HBD2, XBD, Ar2 | 94 |
| 160 | Ar1, HBA3, HBD2, XBD, HBA1 | 174 |
| 161 | Ar1, HBA3, HBD2, XBD, HBA2 | 90 |
| 162 | Ar1, HBA3, HBD2, XBD, HBD1 | 44 |
| 163 | Ar1, HBA3, HBD2, Ar2, HBA1 | 95 |
| 164 | Ar1, HBA3, HBD2, Ar2, HBA2 | 65 |
| 165 | Ar1, HBA3, HBD2, Ar2, HBD1 | 233 |
| 166 | Ar1, HBA3, HBD2, HBA1, HBA2 | 57 |
| 167 | Ar1, HBA3, HBD2, HBA1, HBD1 | 179 |
| 168 | Ar1, HBA3, HBD2, HBA2, HBD1 | 98 |
| 169 | Ar2, HBA1, HBD1, H1, H2 | 69 |
| 170 | Ar2, HBA1, HBD1, H1, H3 | 32 |
| 171 | Ar2, HBA1, HBD1, H1, XBD | 390 |
| 172 | Ar2, HBA1, HBD1, H1, Ar1 | 41 |
| 173 | Ar2, HBA1, HBD1, H1, HBA2 | 131 |
| 174 | Ar2, HBA1, HBD1, H1, HBA3 | 190 |
| 175 | Ar2, HBA1, HBD1, H1, HBD2 | 120 |
| 176 | Ar2, HBA1, HBD1, H2, H3 | 118 |
| 177 | Ar2, HBA1, HBD1, H2, XBD | 146 |
| 178 | Ar2, HBA1, HBD1, H2, Ar1 | 103 |
| 179 | Ar2, HBA1, HBD1, H2, HBA2 | 330 |
| 180 | Ar2, HBA1, HBD1, H2, HBA3 | 153 |
| 181 | Ar2, HBA1, HBD1, H2, HBD2 | 127 |
| 182 | Ar2, HBA1, HBD1, H3, XBD | 97 |
| 183 | Ar2, HBA1, HBD1, H3, Ar1 | 54 |
| 184 | Ar2, HBA1, HBD1, H3, HBA2 | 70 |
| 185 | Ar2, HBA1, HBD1, H3, HBA3 | 52 |
| 186 | Ar2, HBA1, HBD1, H3, HBD2 | 51 |
| 187 | Ar2, HBA1, HBD1, XBD, Ar1 | 73 |
| 188 | Ar2, HBA1, HBD1, XBD, HBA2 | 191 |
| 189 | Ar2, HBA1, HBD1, XBD, HBA3 | 97 |
| 190 | Ar2, HBA1, HBD1, XBD, HBD2 | 86 |
| 191 | Ar2, HBA1, HBD1, Ar1, HBA2 | 127 |
| 192 | Ar2, HBA1, HBD1, Ar1, HBA3 | 459 |
| 193 | Ar2, HBA1, HBD1, Ar1, HBD2 | 114 |
| 194 | Ar2, HBA1, HBD1, HBA2, HBA3 | 104 |
| 195 | Ar2, HBA1, HBD1, HBA2, HBD2 | 141 |
| 196 | Ar2, HBA1, HBD1, HBA3, HBD2 | 68 |
| 197 | Ar2, HBA1, HBD2, H1, H2 | 100 |
| 198 | Ar2, HBA1, HBD2, H1, H3 | 134 |
| 199 | Ar2, HBA1, HBD2, H1, XBD | 51 |
| 200 | Ar2, HBA1, HBD2, H1, Ar1 | 122 |
| 201 | Ar2, HBA1, HBD2, H1, HBA2 | 85 |
| 202 | Ar2, HBA1, HBD2, H1, HBA3 | 84 |
| 203 | Ar2, HBA1, HBD2, H1, HBD1 | 126 |
| 204 | Ar2, HBA1, HBD2, H2, H3 | 15 |
| 205 | Ar2, HBA1, HBD2, H2, XBD | 64 |
| 206 | Ar2, HBA1, HBD2, H2, Ar1 | 65 |
| 207 | Ar2, HBA1, HBD2, H2, HBA2 | 290 |
| 208 | Ar2, HBA1, HBD2, H2, HBA3 | 19 |
| 209 | Ar2, HBA1, HBD2, H2, HBD1 | 36 |
| 210 | Ar2, HBA1, HBD2, H3, XBD | 158 |
| 211 | Ar2, HBA1, HBD2, H3, Ar1 | 140 |
| 212 | Ar2, HBA1, HBD2, H3, HBA2 | 52 |
| 213 | Ar2, HBA1, HBD2, H3, HBA3 | 61 |
| 214 | Ar2, HBA1, HBD2, H3, HBD1 | 51 |
| 215 | Ar2, HBA1, HBD2, XBD, Ar1 | 52 |
| 216 | Ar2, HBA1, HBD2, XBD, HBA2 | 7 |
| 217 | Ar2, HBA1, HBD2, XBD, HBA3 | 194 |
| 218 | Ar2, HBA1, HBD2, XBD, HBD1 | 69 |
| 219 | Ar2, HBA1, HBD2, Ar1, HBA2 | 110 |
| 220 | Ar2, HBA1, HBD2, Ar1, HBA3 | 121 |
| 221 | Ar2, HBA1, HBD2, Ar1, HBD1 | 110 |
| 222 | Ar2, HBA1, HBD2, HBA2, HBA3 | 101 |
| 223 | Ar2, HBA1, HBD2, HBA2, HBD1 | 127 |
| 224 | Ar2, HBA1, HBD2, HBA3, HBD1 | 100 |
| 225 | Ar2, HBA2, HBD1, H1, H2 | 124 |
| 226 | Ar2, HBA2, HBD1, H1, H3 | 133 |
| 227 | Ar2, HBA2, HBD1, H1, XBD | 33 |
| 228 | Ar2, HBA2, HBD1, H1, Ar1 | 191 |
| 229 | Ar2, HBA2, HBD1, H1, HBA1 | 144 |
| 230 | Ar2, HBA2, HBD1, H1, HBA3 | 152 |
| 231 | Ar2, HBA2, HBD1, H1, HBD2 | 96 |
| 232 | Ar2, HBA2, HBD1, H2, H3 | 80 |
| 233 | Ar2, HBA2, HBD1, H2, XBD | 123 |
| 234 | Ar2, HBA2, HBD1, H2, Ar1 | 141 |
| 235 | Ar2, HBA2, HBD1, H2, HBA1 | 74 |
| 236 | Ar2, HBA2, HBD1, H2, HBA3 | 146 |
| 237 | Ar2, HBA2, HBD1, H2, HBD2 | 134 |
| 238 | Ar2, HBA2, HBD1, H3, XBD | 2 |
| 239 | Ar2, HBA2, HBD1, H3, Ar1 | 20 |
| 240 | Ar2, HBA2, HBD1, H3, HBA1 | 20 |
| 241 | Ar2, HBA2, HBD1, H3, HBA3 | 12 |
| 242 | Ar2, HBA2, HBD1, H3, HBD2 | 7 |
| 243 | Ar2, HBA2, HBD1, XBD, Ar1 | 50 |
| 244 | Ar2, HBA2, HBD1, XBD, HBA1 | 174 |
| 245 | Ar2, HBA2, HBD1, XBD, HBA3 | 146 |
| 246 | Ar2, HBA2, HBD1, XBD, HBD2 | 30 |
| 247 | Ar2, HBA2, HBD1, Ar1, HBA1 | 70 |
| 248 | Ar2, HBA2, HBD1, Ar1, HBA3 | 32 |
| 249 | Ar2, HBA2, HBD1, Ar1, HBD2 | 50 |
| 250 | Ar2, HBA2, HBD1, HBA1, HBA3 | 37 |
| 251 | Ar2, HBA2, HBD1, HBA1, HBD2 | 124 |
| 252 | Ar2, HBA2, HBD1, HBA3, HBD2 | 88 |
| 253 | Ar2, HBA2, HBD2, H1, H2 | 150 |
| 254 | Ar2, HBA2, HBD2, H1, H3 | 11 |
| 255 | Ar2, HBA2, HBD2, H1, XBD | 26 |
| 256 | Ar2, HBA2, HBD2, H1, Ar1 | 125 |
| 257 | Ar2, HBA2, HBD2, H1, HBA1 | 85 |
| 258 | Ar2, HBA2, HBD2, H1, HBA3 | 42 |
| 259 | Ar2, HBA2, HBD2, H1, HBD1 | 127 |
| 260 | Ar2, HBA2, HBD2, H2, H3 | 1 |
| 261 | Ar2, HBA2, HBD2, H2, XBD | 44 |
| 262 | Ar2, HBA2, HBD2, H2, Ar1 | 6 |
| 263 | Ar2, HBA2, HBD2, H2, HBA1 | 176 |
| 264 | Ar2, HBA2, HBD2, H2, HBA3 | 114 |
| 265 | Ar2, HBA2, HBD2, H2, HBD1 | 86 |
| 266 | Ar2, HBA2, HBD2, H3, XBD | 87 |
| 267 | Ar2, HBA2, HBD2, H3, Ar1 | 133 |
| 268 | Ar2, HBA2, HBD2, H3, HBA1 | 76 |
| 269 | Ar2, HBA2, HBD2, H3, HBA3 | 6 |
| 270 | Ar2, HBA2, HBD2, H3, HBD1 | 158 |
| 271 | Ar2, HBA2, HBD2, XBD, Ar1 | 55 |
| 272 | Ar2, HBA2, HBD2, XBD, HBA1 | 60 |
| 273 | Ar2, HBA2, HBD2, XBD, HBA3 | 1 |
| 274 | Ar2, HBA2, HBD2, XBD, HBD1 | 53 |
| 275 | Ar2, HBA2, HBD2, Ar1, HBA1 | 29 |
| 276 | Ar2, HBA2, HBD2, Ar1, HBA3 | 100 |
| 277 | Ar2, HBA2, HBD2, Ar1, HBD1 | 57 |
| 278 | Ar2, HBA2, HBD2, HBA1, HBA3 | 94 |
| 279 | Ar2, HBA2, HBD2, HBA1, HBD1 | 7 |
| 280 | Ar2, HBA2, HBD2, HBA3, HBD1 | 85 |
| 281 | Ar2, HBA3, HBD1, H1, H2 | 52 |
| 282 | Ar2, HBA3, HBD1, H1, H3 | 126 |
| 283 | Ar2, HBA3, HBD1, H1, XBD | 69 |
| 284 | Ar2, HBA3, HBD1, H1, Ar1 | 64 |
| 285 | Ar2, HBA3, HBD1, H1, HBA1 | 145 |
| 286 | Ar2, HBA3, HBD1, H1, HBA2 | 27 |
| 287 | Ar2, HBA3, HBD1, H1, HBD2 | 150 |
| 288 | Ar2, HBA3, HBD1, H2, H3 | 190 |
| 289 | Ar2, HBA3, HBD1, H2, XBD | 102 |
| 290 | Ar2, HBA3, HBD1, H2, Ar1 | 153 |
| 291 | Ar2, HBA3, HBD1, H2, HBA1 | 105 |
| 292 | Ar2, HBA3, HBD1, H2, HBA2 | 450 |
| 293 | Ar2, HBA3, HBD1, H2, HBD2 | 66 |
| 294 | Ar2, HBA3, HBD1, H3, XBD | 126 |
| 295 | Ar2, HBA3, HBD1, H3, Ar1 | 87 |
| 296 | Ar2, HBA3, HBD1, H3, HBA1 | 330 |
| 297 | Ar2, HBA3, HBD1, H3, HBA2 | 130 |
| 298 | Ar2, HBA3, HBD1, H3, HBD2 | 140 |
| 299 | Ar2, HBA3, HBD1, XBD, Ar1 | 181 |
| 300 | Ar2, HBA3, HBD1, XBD, HBA1 | 678 |
| 301 | Ar2, HBA3, HBD1, XBD, HBA2 | 97 |
| 302 | Ar2, HBA3, HBD1, XBD, HBD2 | 129 |
| 303 | Ar2, HBA3, HBD1, Ar1, HBA1 | 400 |
| 304 | Ar2, HBA3, HBD1, Ar1, HBA2 | 134 |
| 305 | Ar2, HBA3, HBD1, Ar1, HBD2 | 76 |
| 306 | Ar2, HBA3, HBD1, HBA1, HBA2 | 770 |
| 307 | Ar2, HBA3, HBD1, HBA1, HBD2 | 116 |
| 308 | Ar2, HBA3, HBD1, HBA2, HBD2 | 116 |
| 309 | Ar2, HBA3, HBD2, H1, H2 | 58 |
| 310 | Ar2, HBA3, HBD2, H1, H3 | 68 |
| 311 | Ar2, HBA3, HBD2, H1, XBD | 23 |
| 312 | Ar2, HBA3, HBD2, H1, Ar1 | 92 |
| 313 | Ar2, HBA3, HBD2, H1, HBA1 | 153 |
| 314 | Ar2, HBA3, HBD2, H1, HBA2 | 148 |
| 315 | Ar2, HBA3, HBD2, H1, HBD1 | 83 |
| 316 | Ar2, HBA3, HBD2, H2, H3 | 130 |
| 317 | Ar2, HBA3, HBD2, H2, XBD | 95 |
| 318 | Ar2, HBA3, HBD2, H2, Ar1 | 200 |
| 319 | Ar2, HBA3, HBD2, H2, HBA1 | 143 |
| 320 | Ar2, HBA3, HBD2, H2, HBA2 | 103 |
| 321 | Ar2, HBA3, HBD2, H2, HBD1 | 100 |
| 322 | Ar2, HBA3, HBD2, H3, XBD | 123 |
| 323 | Ar2, HBA3, HBD2, H3, Ar1 | 190 |
| 324 | Ar2, HBA3, HBD2, H3, HBA1 | 200 |
| 325 | Ar2, HBA3, HBD2, H3, HBA2 | 130 |
| 326 | Ar2, HBA3, HBD2, H3, HBD1 | 86 |
| 327 | Ar2, HBA3, HBD2, XBD, Ar1 | 168 |
| 328 | Ar2, HBA3, HBD2, XBD, HBA1 | 112 |
| 329 | Ar2, HBA3, HBD2, XBD, HBA2 | 126 |
| 330 | Ar2, HBA3, HBD2, XBD, HBD1 | 103 |
| 331 | Ar2, HBA3, HBD2, Ar1, HBA1 | 137 |
| 332 | Ar2, HBA3, HBD2, Ar1, HBA2 | 78 |
| 333 | Ar2, HBA3, HBD2, Ar1, HBD1 | 179 |
| 334 | Ar2, HBA3, HBD2, HBA1, HBA2 | 29 |
| 335 | Ar2, HBA3, HBD2, HBA1, HBD1 | 71 |
| 336 | Ar2, HBA3, HBD2, HBA2, HBD1 | 123 |
| **Total Hit** | | **41248** |

| **SL** | **Name** | **Pharmacophore fit score** | **Number of Conformers** | **RMSD** | **Matching Featurres** |
| --- | --- | --- | --- | --- | --- |
| 01 | ZINC94272748 | 96.90 | 25 | 0.60 | 9 |
| 02 | ZINC79046938 | 95.91 | 25 | 0.49 | 9 |
| 03 | ZINC05925939 | 87.55 | 11 | 0.07 | 8 |
| 04 | ZINC59928516 | 86.96 | 25 | 0,48 | 8 |
| 05 | ZINC13831249 | 86.88 | 25 | 0.22 | 8 |
| 06 | ZINC91511779 | 85.87 | 16 | 0.29 | 8 |
| 07 | ZINC38658035 | 85.26 | 25 | 0.52 | 8 |
| 08 | ZINC05008863 | 84.97 | 26 | 0.50 | 8 |
| 09 | ZINC06068955 | 68.38 | 25 | 0.63 | 6 |
| 10 | ZINC34940571 | 68.06 | 19 | 0.38 | 6 |
| 11 | ZINC14457254 | 68.00 | 25 | 0.40 | 6 |
| 12 | ZINC34973221 | 67.96 | 22 | 0.38 | 6 |
| 13 | ZINC67981677 | 67.92 | 25 | 0.46 | 6 |
| 14 | ZINC67851844 | 67.92 | 25 | 0.46 | 6 |
| 15 | ZINC94993264 | 67.91 | 16 | 0.54 | 6 |
| 16 | ZINC06462060 | 67.85 | 25 | 0.62 | 6 |
| 17 | ZINC06462054 | 67.85 | 25 | 0.62 | 6 |
| 18 | ZINC06462046 | 67.85 | 25 | 0.62 | 6 |
| 19 | ZINC06462053 | 67.85 | 25 | 0.62 | 6 |
| 20 | ZINC06462055 | 67.83 | 22 | 0.62 | 6 |
| 21 | ZINC06915040 | 67.83 | 25 | 0.60 | 6 |
| 22 | ZINC67879406 | 67.83 | 25 | 0.46 | 6 |
| 23 | ZINC06822171 | 67.82 | 25 | 0.62 | 6 |
| 24 | ZINC06462052 | 67.82 | 25 | 0.62 | 6 |
| 25 | ZINC06462048 | 67.80 | 25 | 0.62 | 6 |
| 26 | ZINC06462059 | 67.79 | 25 | 0.62 | 6 |
| 27 | ZINC63848565 | 67.79 | 25 | 0.42 | 6 |
| 28 | ZINC05071941 | 67.79 | 25 | 0.57 | 6 |
| 29 | ZINC06462051 | 67.78 | 25 | 0.62 | 6 |
| 30 | ZINC09645631 | 67.78 | 25 | 0.61 | 6 |
| 31 | ZINC20689956 | 67.74 | 25 | 0.54 | 6 |
| 32 | ZINC09090302 | 67.71 | 25 | 0.53 | 6 |
| 33 | ZINC06462193 | 67.69 | 25 | 0.73 | 6 |

**Table S2: 2^nd^ Round of Virtual Screening Result: 33 hits from 41,248 compounds library**

**Table S3:** Physicochemical properties analysis of the control drug (Tamoxifen) and the top four hit compounds

| **Compound** | **MW**  **(g/mol)** | **TPSA (Å²) and Molar Refractivity (MR)** | **H. Bond** | **Rotatabole Bonds/ Surface Area** | **Lipophilicity**  **(Consensus Log P_o/w_)** | **Water Solubility**  **(Log *S* (ESOL))** | **Pharmacokinetics** | **Drug likeness** |
| --- | --- | --- | --- | --- | --- | --- | --- | --- |
| Tamoxifen (Control) | 371.51 | TPSA: 12.47 | Acceptor: 8 | 8 | 5.77 | -6.59 | GI absorption: Low | Lipinski: Yes, 1 violation  Ghose: No, 1 violation  Veber: Yes  Egan: No, 1 violation  Muegge: No, 1 violation  Bioavailability Score: 0.55 |
|  |  | MR: 119.72 | Donor: 2 | 168.649 |  |  | BBB permeant: no |  |
| ZINC94272748 | 305.74 | TPSA:102.02 | Acceptor: 7 | 3 | 2.32 | -3.95 | GI absorption: High | Lipinski: Yes  Ghose: Yes  Veber: Yes  Egan: Yes  Muegge: Yes  Bioavailability Score: 0.55 |
|  |  | MR: 74.55 | Donor: 1 | 122.969 |  |  | BBB permeant: no |  |
| ZINC79046938 | 318.78 | TPSA:120.45 | Acceptor: 5 | 6 | 1.79 | -3.10 | GI absorption: High | Lipinski: Yes  Ghose: Yes  Veber: Yes  Egan: Yes  Muegge: Yes  Bioavailability Score: 0.55 |
|  |  | MR: 82.57 | Donor: 2 | 125.206 |  |  | BBB permeant: no |  |
| ZINC05925939 | 267.32 | TPSA: 64.25 | Acceptor: 3 | 4 | 3.17 | -4.03 | GI absorption: High | Lipinski: Yes  Ghose: Yes  Veber: Yes  Egan: Yes  Muegge: Yes  Bioavailability Score: 0.55 |
|  |  | MR: 78.92 | Donor: 2 | 118.294 |  |  | BBB permeant: no |  |
| ZINC59928516 | 350.80 | TPSA: 89.13 | Acceptor: 5 | 4 | 3.74 | -5.03 | GI absorption: High | Lipinski: Yes  Ghose: Yes  Veber: Yes  Egan: Yes  Muegge: Yes  Bioavailability Score: 0.55 |
|  |  | MR: 87.16 | Donor: 1 | 140.645 |  |  | BBB permeant: no |  |

**Table S4:** Contact amino acid residues of top four hit compounds and the control drug (Tamoxifen)

| **Ligands** | **Contact Residues** |
| --- | --- |
| Control (Tamoxifen) | GLU332 TRP335 MET473 LEU476 LEU477 MET479 CYS481 VAL484 VAL485 PRO486 VAL487 TYR488 ASP489 |
| ZINC94272748 | MET295 LEU298 THR299 LEU301 ALA302 GLU305 TRP335 MET336 LEU339 MET340 LEU343 ARG346 PHE356 ILE373 ILE376 PHE377 LEU380 GLY472 HIS475 LEU476 VAL487 |
| ZINC79046938 | MET295 LEU298 THR299 LEU301 ALA302 GLU305 MET336 LEU339 MET340 LEU343 ARG346 PHE356 ILE373 ILE376 PHE377 LEU380 GLY472 HIS475 LEU476 VAL484 |
| ZINC05925939 | MET295 LEU298 THR299 LEU301 ALA302 GLU305 MET336 LEU339 MET340 LEU343 PHE356 ILE373 ILE376 PHE377 LEU380 GLY472 HIS475 LEU476 MET479 |
| ZINC59928516 | GLU276 PRO277 PRO278 HIS279 VAL280 GLU305 LEU306 HIS308 MET309 VAL338 LEU339 GLY342 ARG346 PHE356 ALA357 PRO358 TYR397 LYS401 |

**Table S5: Molecular Dynamics Simulations:** Properties of the control and the top four compounds.

| **SL** | **Name** | **Matrices** | **Highest** | **Lowest** | **AV** |
| --- | --- | --- | --- | --- | --- |
| 01 | Tamoxifen (control) | RMSD (Å) | 0.844 | 0 | 0.46445 |
|  |  | RMSF (Å) | 1.984 (Atom no: 3) | 0.997 (Atom no: 14) | 1.48435 |
|  |  | RG (Å) | 4.055 | 3.887 | 3.973084 |
|  |  | SASA (Å^2^) | 29.341 | 0 | 3.39943 |
|  |  | MolSA (Å^2^) | 242.372 | 234.109 | 237.7174 |
|  |  | PSA (Å^2^) | 198.137 | 185.669 | 191.56 |
|  |  | IntraHB | 1 | 0 | 0.994012 |
| 02 | ZINC94272748 | RMSD (Å) | 1.727 | 0 | 1.275028 |
|  |  | RMSF (Å) | 3.326(Atom no:15) | 0.775 (Atom no: 5) | 1.63705 |
|  |  | RG (Å) | 4.068 | 3.327 | 3.748928 |
|  |  | SASA (Å^2^) | 17.46 | 0 | 1.058401 |
|  |  | MolSA (Å^2^) | 259.177 | 235.557 | 250.6109 |
|  |  | PSA (Å^2^) | 162.41 | 144.378 | 153.1312 |
| 03 | ZINC79046938 | RMSD (Å) | 1.726 | 0 | 0.699878 |
|  |  | RMSF (Å) | 2.568(Atom no:14) | 1.08 (Atom no:2) | 1.5681 |
|  |  | RG (Å) | 4.525 | 3.663 | 4.122775 |
|  |  | SASA (Å^2^) | 26.647 | 0 | 4.328577 |
|  |  | MolSA (Å^2^) | 286.636 | 265.906 | 274.5362 |
|  |  | PSA (Å^2^) | 181.607 | 146.169 | 168.0278 |
| 04 | ZINC05925939 | RMSD (Å) | 0.872 | 0 | 0.457712 |
|  |  | RMSF (Å) | 1.37(Atom no: 18) | 0.636(Atom no: 4) | 0.8984 |
|  |  | RG (Å) | 3.914 | 3.714 | 3.809431 |
|  |  | SASA (Å^2^) | 15.508 | 0 | 2.668359 |
|  |  | MolSA (Å^2^) | 272.891 | 262.424 | 267.8755 |
|  |  | PSA (Å^2^) | 160.572 | 149.282 | 154.4766 |
| 05 | ZINC59928516 | RMSD (Å) | 3.268 | 0 | 1.843805 |
|  |  | RMSF (Å) | 3.27 (Atom no: 20) | 1.017 (Atom no: 9) | 1.88613 |
|  |  | RG (Å) | 4.483 | 3.426 | 4.013935 |
|  |  | SASA (Å^2^) | 19.732 | 0 | 2.353914 |
|  |  | MolSA (Å^2^) | 305.225 | 261.909 | 294.0014 |
|  |  | PSA (Å^2^) | 136.993 | 113.551 | 124.0335 |
